# Sodium‐glucose co‐transporter 2 inhibitors and obesity‐associated cancers in people with type 2 diabetes: A real‐world observational study

**DOI:** 10.1111/dom.70562

**Published:** 2026-02-26

**Authors:** Testimony Ipaye, Jonathan Goldney, Thomas Yates, Francesco Zaccardi, Kamlesh Khunti, Elina Akalestou, Melanie J. Davies, Karen Brown, Dimitris Papamargaritis

**Affiliations:** ^1^ NIHR Leicester Biomedical Research Centre University Hospitals of Leicester NHS Trust Leicester UK; ^2^ Leicester Diabetes Centre, Leicester General Hospital University of Leicester Leicester UK; ^3^ Leicester Cancer Research Centre University of Leicester Leicester UK; ^4^ Leicester Real World Evidence Unit Leicester UK; ^5^ Division of Cardiovascular Sciences, College of Life Sciences University of Leicester Leicester UK; ^6^ Section of Cell Biology and Functional Genomics, Division of Diabetes, Endocrinology and Metabolism, Department of Metabolism, Digestion and Reproduction Imperial College London London UK

**Keywords:** cancer, obesity, overweight, SGLT‐2i, type 2 diabetes

## Abstract

**Aims:**

To investigate the association between sodium‐glucose co‐transporter 2 inhibitors (SGLT‐2i) and obesity‐associated cancers (OAC) in individuals with type 2 diabetes (T2D).

**Materials and Methods:**

This retrospective cohort study used data from the TriNetX US Collaborative Network. Individuals with T2D initiating SGLT‐2i were compared with those initiating dipeptidyl peptidase‐4 inhibitors (DPP‐4i) using 1:1 propensity score matching to account for key confounders. Individuals with prior cancer or cancer diagnosed within 6 months after treatment initiation were excluded. Hazard ratios (HRs) and 95% confidence intervals (CIs) for composite OAC, individual OAC types, and all‐cancer outcomes were estimated using Cox proportional hazards models. Analyses were conducted at the overall cohort, stratified by overweight/obesity status and across individual SGLT‐2i agents.

**Results:**

Initiation of SGLT‐2i was not associated with composite OACs compared with DPP‐4i in the overall population or across overweight/obesity status. A lower rate of renal cancer was also observed in the overall population (HR 0.78; 95% CI 0.67–0.92) and among individuals with overweight/obesity (0.79; 0.66–0.93), while a higher rate of thyroid cancer was observed in the overall population (1.37; 1.06–1.76). Modest reductions in the all‐cancer outcome was observed in the overall population (0.96; 0.93–0.99) and among individuals with (0.95; 0.91–0.98) and without (0.88; 0.78–0.99) overweight/obesity.

**Conclusion:**

In this large real‐world study of individuals with T2D, initiation of SGLT‐2i was not associated with higher or lower rates of OAC compared with DPP‐4i. However, initiation of SGLT‐2i was associated with a modestly lower rate of the all‐cancer outcome, the clinical significance of which remains unclear.

## 1 INTRODUCTION

The rising global prevalence of obesity and type 2 diabetes (T2D), both linked to increased cancer risk and cancer‐related mortality, has contributed to a worldwide surge in cancer rates.^1^, ^2^ The link between overweight/obesity and 13 specific types of cancer known as obesity‐associated cancers (OAC) is well established^3^ and has been proposed to account for a 35% increase in cancer‐related deaths between 2010 and 2019.^4^

The relationship between T2D, obesity, and OAC is thought to be driven by shared biological mechanisms, including hyperglycaemia, hyperinsulinemia, and chronic low‐grade inflammation.^1^, ^5^ As obesity is a modifiable risk factor for both T2D and cancer, interventions that improve metabolic health and promote sustained weight loss may plausibly influence OAC risk.

Weight loss‐focused interventions such as bariatric surgery and glucagon‐like peptide‐1 receptor agonists (GLP‐1 RAs) have been associated with improved glycaemic control and reduced OAC risk in individuals with obesity and T2D.^6^ However, despite their efficacy, both approaches face important limitations in real‐world implementation. Bariatric surgery is invasive and constrained by access, cost, and healthcare capacity,^7^, ^8^ while GLP‐1 RA use is limited by high cost, restricted reimbursement, adverse effects, and ongoing supply challenges.^9^, ^10^ These barriers highlight the need to explore more widely accessible pharmacological strategies for cancer prevention in individuals with obesity and T2D.

Sodium‐glucose co‐transporter‐2 inhibitors (SGLT‐2i), an oral treatment for T2D, are a class of glucose‐lowering medications that improve glucose levels by inhibiting glucose reabsorption in the proximal tubules of the kidney (promoting urinary glucose excretion).^11^, ^12^ First approved over a decade ago and now available in generic formulations, SGLT‐2i also result in 2–3 kg mean weight loss (WL) in people with T2D.^13^, ^14^ Beyond glycaemic control and WL, SGLT‐2i have also demonstrated cardio‐ and reno‐protective benefits in people with and without T2D (especially in those with heart failure and chronic kidney disease).^15^, ^16^ Additionally, preclinical studies have shown that SGLT‐2i may have anti‐cancer properties.^17^, ^18^, ^19^, ^20^, ^21^, ^22^

This study investigates whether SGLT‐2i are associated with a reduced rate of OAC, as compared to dipeptidyl‐peptidase‐4 inhibitors (DPP‐4i), a weight‐neutral alternative oral treatment for T2D, with no known association with cancer risk,^23^ in individuals with T2D.

## 2 MATERIALS AND METHODS

This study followed the Reporting of studies Conducted using Observational Routinely‐collected health Data (RECORD) guidelines presented in Data S1, Supporting Information.

### 2.1 Data source

This study used data from the US Collaborative Network in the TriNetX federated research database^24^ to access deidentified pseudo‐anonymised electronic health records collated across numerous health care organisations (HCOs) in the United States. The US Collaborative network contains clinical data including diagnostic codes, demographic data, medication, procedures and laboratory results.^24^ Data are available via an in‐built TriNetX analysis platform for real‐time research use that provides aggregate results to researchers. Data protection laws applicable to the contributing HCOs, including the US Health Insurance Portability and Accountability Act, were observed during data processing and transmission. Due to the anonymisation process and utilisation of routinely collected data, ethical approval and consent were not required.

### 2.2 Study population

**FIGURE 1:**
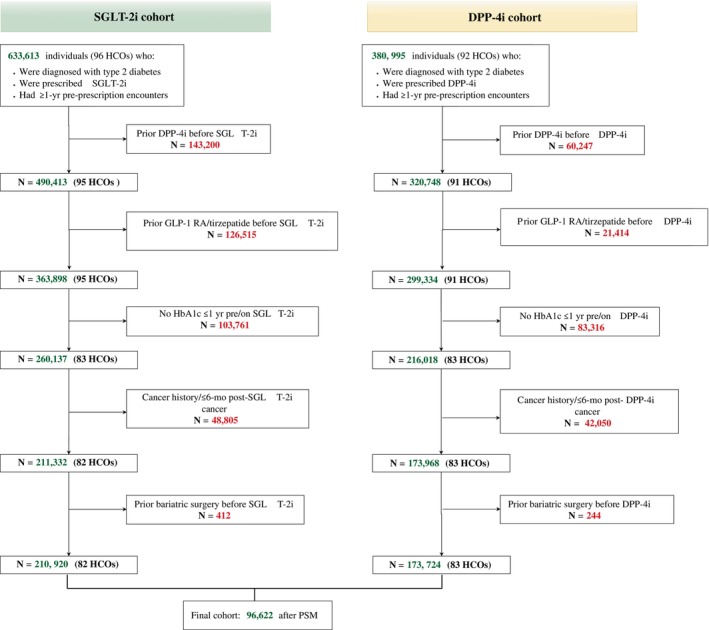
Flow diagram reporting the proportion of missing data. Note that all numbers represent approximations, due to the live nature of the TriNetX platform, the number of HCOs contributing data to each request are subject to change. Understanding the cohort definition requires several iterative requests for data from the platform and contributing HCOs and cannot be undertaken simultaneously due to platform limitations. DPP‐4i: Dipeptidyl Peptidase‐4 inhibitors; HCOs: Health Care Organizations; GLP1‐RA: Glucagon‐like Peptide‐1 Receptor Agonists; PSM: Propensity Score Matching; SGLT‐2i: Sodium–Glucose Cotransporter‐2 inhibitors.

This study compared outcomes in people with T2D who received a first‐ever prescription for any SGLT‐2i with those receiving a first‐ever prescription for any DPP‐4i. Individuals were excluded if, prior to the index date, they had type 1 diabetes; any previous cancer diagnosis occurring before or within 6 months after the index date, to minimise reverse causality or detection bias; or if they had any exposure to GLP‐1 RAs or tirzepatide before the index date, as these medications are not indicated for use with DPP‐4i due to shared mechanistic pathways. Additional exclusions included no recorded body mass index (BMI) or HbA1c measurement in the past 12 months (to ensure data availability for covariate balancing), history of bariatric surgery prior to index date, and first HCO visit >12 months prior to index date to improve accuracy of medical record (Figure 1). The index date was defined as the date of first prescription for either SGLT‐2i or DPP‐4i. DPP‐4i were selected as comparators because they are commonly prescribed oral glucose‐lowering therapies, are weight‐neutral,^25^ and are not known to be associated with cancer.^23^

### 2.3 Outcomes

We investigated the rate of first occurrence of all major OAC, both as a composite outcome (our primary outcome) and as individual cancer types (constituting secondary outcomes), including breast, colorectal, uterine, gallbladder, gastric cardia, renal, liver, multiple myeloma, oesophageal, ovarian, pancreatic, and thyroid cancers.^3^ Meningioma was excluded from analysis as it is predominantly benign. OAC were specified a priori as the primary outcome, reflecting their established biological links to obesity, metabolic dysfunction, and T2D. For completeness, and due to potential weight‐independent anti‐cancer effects of SGLT‐2i,^17^, ^18^, ^19^, ^20^, ^21^, ^22^ we also assessed the rate of first occurrence of any cancer as a secondary composite outcome. The clinical codes used to identify these outcomes are presented in Table S1.

### 2.4 Subgroup analyses

To further understand if the association of SGLT‐2i with cancer risk varies with overweight/obesity status, we undertook a subgroup analysis comparing SGLT‐2i to DPP‐4i separately in individuals with and without overweight/obesity (defined by BMI ≥25 kg/m^2^ in the 12 months prior to index date). We also performed an additional subgroup analysis investigating whether associations with composite‐cancer outcomes vary with the individual SGLT‐2i agents, including (a) canagliflozin; (b) dapagliflozin; (c) empagliflozin; (d) ertugliflozin.

### 2.5 Confounding factors

Potential confounding factors from HCO records collected on the TriNetX platform were identified up to 12 months prior to initiation of SGLT‐2i or DPP‐4i (i.e., the index date). These confounding factors included demographics: age at index, sex (male/female/unknown), race (American Indian or Alaska native/Asian/Black or African American/Hispanic or Latino/Native Hawaiian or Other Pacific Islander/Not Hispanic or Latino/other race/unknown race/unknown Ethnicity/White); Anthropometrics: BMI in categories of 5 kg/m^2^ between 25 and 55 kg/m^2^, HbA1c in categories of 1% between 6.5% and 11.5%; Medications: metformin, insulin, sulfonylureas, antihypertensives, lipid modifying agents; Past medical history: ischemic heart diseases, hypertensive diseases, T2D with kidney complications, T2D with neurological complications, T2D with ophthalmic complications, cerebrovascular diseases, peripheral vascular disease, atherosclerosis, nicotine dependence, alcohol dependence, alcohol‐related disorders, personal history of nicotine dependence; Social factors: persons with potential health hazards related to socio‐economic and psychosocial circumstances. The clinical codes used to identify confounding factors are presented in Table S2.

### 2.6 Sensitivity analyses

To explore differences in findings between the present results and previously published results, additional sensitivity analyses were undertaken: (1) an analysis excluding individuals with GLP‐1 RA or tirzepatide use following SGLT‐2i/DPP‐4i initiation, to address the potential confounding by GLP‐1‐based therapies which may have anti‐cancer effects, as SGLT‐2i users may be more likely to receive GLP‐1‐based therapies after index due to these agents not being indicated for use with DPP‐4i; and (2) an analysis restricted to individuals with repeated SGLT‐2i or DPP‐4i use recorded at least 6 months after initial initiation, to exclude individuals with early discontinuation.

### 2.7 Statistical analysis

We investigated relative rates of composite‐OAC, individual OACs, and all‐cancer outcomes in the SGLT‐2i cohort as compared to the DPP‐4i cohort. Baseline characteristics (before and after propensity score matching) for the SGLT‐2i cohort and the DPP‐4i cohort were calculated separately. Continuous variables were reported as means and standard deviations (SDs) while categorical variables were reported as numbers and percentages. Propensity score matching was performed using a 1:1 “greedy nearest neighbour” approach with a calliper width of 0.1 SDs, adjusting for the previously identified confounding variables. Standardised mean difference (SMD) in matched covariates was calculated across SGLT‐2i and DPP‐4i cohorts, with SMD <0.1 considered to be well‐matched.

The index date was defined as the first record of SGLT‐2i or DPP‐4i at any HCO in the TriNetX network, with data censored at the occurrence of an outcome, or at the most recently recorded visit to a HCO in the TriNetX network before the date of analysis (18 January 2026). The Cox proportional‐hazards model was used to assess the relative rates of outcomes (composite and individual OACs: breast, colorectal, corpus uteri, gallbladder, gastric cardia, renal, liver, multiple myeloma, oesophageal, ovarian, pancreatic, and thyroid; and an all‐cancer composite outcome), comparing SGLT‐2i to DPP‐4i.

For subgroup analyses, cohort matching and all analyses were repeated for individuals with and without overweight/obesity separately, and for individuals initiating each individual SGLT‐2i (canagliflozin/dapagliflozin/empagliflozin/ertugliflozin) separately (without BMI criteria). Similarly, matching and Cox models were repeated for sensitivity analyses, firstly excluding individuals with any GLP‐1 RA or tirzepatide prescription after index, and separately including only individuals persistent to the medication exposure for >6 months.

All HRs were reported with 95% confidence intervals (CIs). Interaction *p*‐values across overweight/obesity status and exposures with respect to cancer risk were calculated manually by comparing the HR and 95% CI in the sub‐analyses with versus without overweight/obesity.^26^, ^27^ For HRs, an upper bound 95% CI <1, or lower bound 95% CI >1, was considered statistically significant; for interactions, a *p*‐value <0.05 was considered statistically significant.

Analyses were conducted on the TriNetX platform, utilising R's survival package (version 3.2‐3) for Cox regression modelling. Proportionality was assessed visually with KM curves and also by utilising Schoenfeld residuals (Figure S1). To protect health information, results with counts less than 10 were suppressed. Figures were created and formatted in RStudio v4.4.2 using the *forest plot* package.

## 3 RESULTS

### 3.1 Baseline characteristics

Table 1 presents the number of individuals identified across the matched SGLT‐2i and DPP‐4i cohorts in the overall population with T2D: 92 052 individuals were matched in each treatment group (mean age at index: 62.9 years for SGLT‐2i vs. 63.0 years for DPP‐4i; 47.6% vs. 48.0% women respectively). Demographics, BMI, HbA1c, medication and past medical history were well matched (SMD <0.1). Baseline characteristics before propensity score matching are reported in Table S3.

**TABLE 1:** Baseline characteristics after propensity score matching from primary analysis.

		SGLT‐2i versus DPP‐4i		
		Exposure (SGLT‐2i)	Comparator (DPP‐4i)	SMD
Number		92 052	92 052	‐
Age at index date (years)		62.9 (12.4)	63.0 (14.2)	.007
Sex	Female	43 814 (47.6)	44 146 (48.0)	.007
	Male	48 182 (52.3)	47 851 (52.0)	.007
	Unknown sex	56 (0.1)	55 (0.1)	.000
Race	American Indian or Alaska Native	522 (0.6)	542 (0.6)	.003
	Asian	9174 (10)	9034 (9.8)	.005
	Black or African American	18 647 (20.3)	18 865 (20.5)	.006
	Native Hawaiian or Other Pacific Islander	979 (1.1)	965 (1.1)	.001
	White	53 635 (58.3)	53 626 (58.3)	.000
	Other race	3612 (3.9)	3553 (3.9)	.003
	Unknown race	5483 (6.0)	5467 (5.9)	.001
Ethnicity	Hispanic or Latino	7657 (8.3)	7508 (8.2)	.006
	Not Hispanic or Latino	67 965 (73.8)	68 065 (73.9)	.002
	Unknown Ethnicity	16 430 (17.9)	16 479 (17.9)	.001
BMI kg/m^2^		32.4 (7.6)	32.2 (7.7)	.020
BMI categories (kg/m^2^)	<25	17 980 (19.5)	17 810 (19.4)	.005
	25–30	33 568 (36.5)	33 320 (36.2)	.006
	30–35	32 524 (35.3)	32 596 (35.4)	.002
	35–40	20 914 (22.7)	21 166 (23)	.007
	40–45	11 221 (12.2)	11 400 (12.4)	.006
	45–50	5389 (5.9)	5386 (5.9)	.000
	50–55	2427 (2.6)	2466 (2.7)	.003
	>55	1586 (1.7)	1634 (1.8)	.004
HbA1c (%)		8.2 (1.9)	8.2 (1.9)	.015
HbA1c categories (%)	<6.5	22 396 (24.3)	22 621 (24.6)	.004
	6.5–7.5	38 978 (42.3)	38 784 (42.1)	.003
	7.5–8.5	30 988 (33.7)	30 837 (33.5)	.000
	8.5–9.5	18 612 (20.2)	18 628 (20.2)	.001
	9.5–10.5	11 096 (12.1)	11 132 (12.1)	.000
	10.5–11.5	7149 (7.8)	7157 (7.8)	.005
	>11.5	9004 (9.8)	8865 (9.6)	.004
Past medical history	Hypertensive diseases	71 633 (77.8)	71 790 (78.0)	.004
	Ischemic heart diseases	22 734 (24.7)	23 142 (25.1)	.010
	Type 2 diabetes mellitus with kidney complications	18 238 (19.8)	18 436 (20.0)	.005
	Type 2 diabetes mellitus with neurological complications	13 066 (14.2)	13 202 (14.3)	.004
	Nicotine dependence	10 083 (11.0)	10 054 (10.9)	.001
	Alcohol dependence	967 (1.1)	983 (1.1)	.002
	Cerebrovascular diseases	9504 (10.3)	9628 (10.5)	.004
	Type 2 diabetes mellitus with ophthalmic complications	4787 (5.2)	4762 (5.2)	.001
	Atherosclerosis	4715 (5.1)	4777 (5.2)	.003
	Personal history of nicotine dependence	13 046 (14.2)	13 364 (14.5)	.010
	Peripheral vascular disease, unspecified	4471 (4.9)	4652 (5.1)	.009
	Alcohol related disorders	2216 (2.4)	2280 (2.5)	.005
Medications	Metformin	53 680 (58.3)	53 136 (57.7)	.012
	Insulin	35 785 (38.9)	36 120 (39.2)	.007
	Antihypertensives	13 904 (15.1)	14 109 (15.3)	.006
	Sulfonylureas	22 203 (24.1)	22 146 (24.1)	.001
	Lipid modifying agents	60 411 (65.6)	60 275 (65.5)	.003
	Bupropion	2787 (3.0)	2648 (2.9)	.009
	Topiramate	906 (1.0)	1037 (1.1)	.014
	Phentermine	279 (0.3)	217 (0.2)	.013
	Naltrexone	219 (0.2)	187 (0.2)	.007
	Orlistat	15 (0.0)	10 (0.0)	.005
Persons with potential health hazards related to socioeconomic and psychosocial circumstances		2933 (3.2)	2876 (3.1)	.004

*Note*: Data are shown as mean (SD) for continuous variables and number (%) for categorical variables. Orlistat, bupropion, naltrexone, and phentermine, topiramate were not included as propensity score matching covariates but are presented for additional information. It was not possible on the TriNetX network to look at specific combinations of these medications which are used for obesity, that is, bupropion and naltrexone are presented separately.

Abbreviations: BMI, body mass index; DPP‐4i, dipeptidyl peptidase‐4 inhibitors; SGLT‐2i, sodium‐glucose co‐transporter 2 inhibitors; SMD, standardised mean difference.

The mean follow‐up time (SD) in the overall T2D cohort used in the primary analysis was 1343 (945) days for the SGLT‐2i group and 1367 (994) days for the DPP‐4i cohort (Table S4).

### 3.2 Primary analysis

**FIGURE 2:**
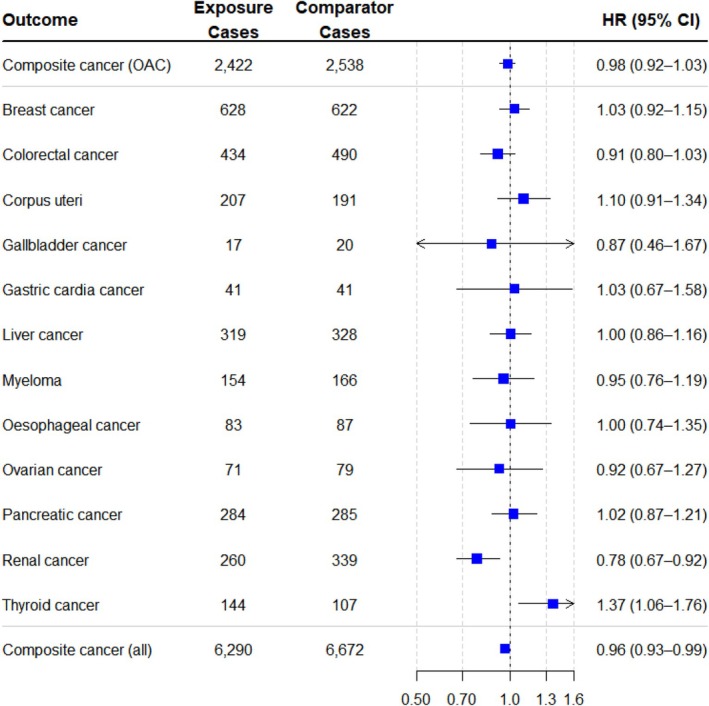
Hazard ratio of obesity‐associated cancers in individuals with type 2 diabetes treated with SGLT‐2i, as compared with DPP‐4i. Number of individuals per cohort: 92 052. HR: Hazard ratio; CI: Confidence interval; OAC: Obesity‐associated cancer.

Figure 2 illustrates the relative rates of (i) composite OAC, (ii) individual OAC types, (iii) composite all‐cancer with SGLT‐2i versus DPP‐4i in the overall T2D population. For the primary outcome, SGLT‐2i were not associated with a significantly higher or lower rate of composite OAC as compared to DPP‐4i (HR: 0.98; 95% CI 0.92–1.03), with 2422 (2.63%) events in the SGLT‐2i group versus 2538 (2.76%) events in the DPP‐4i group.

When investigating individual OAC types as secondary outcomes, SGLT‐2i were associated with lower rates of renal cancer (0.78; 0.67–0.92). No significant differences were observed in the rates of breast (1.03; 0.92–1.15), colorectal (0.91; 0.80–1.03), corpus uteri (1.10; 0.91–1.34), gallbladder (0.87; 0.46–1.67), gastric cardia (1.03; 0.67–1.58), liver (1.00; 0.86–1.16), myeloma (0.95; 0.76–1.19), oesophageal (1.00; 0.74–1.35), ovarian (0.92; 0.67–1.27), or pancreatic (1.02; 0.87–1.21) cancers between the treatment groups. An increased rate of thyroid cancer was associated with SGLT‐2i (1.37; CI 1.06–1.76).

For the broader all‐cancer composite secondary outcome (any incident cancer), SGLT‐2i was associated with a modest but statistically significant difference in rate compared to DPP‐4i (0.96; 0.93–0.99).

### 3.3 Overweight/obesity subgroups

Among individuals with T2D and overweight/obesity, 73 970 individuals were matched per cohort (baseline characteristics presented in Table S5). Among individuals with T2D without overweight/obesity, 10 316 individuals were matched in each cohort. For both subgroups, baseline characteristics were well matched (SMD <0.1; Table S5) between treatment groups. In the overweight/obesity cohort, the mean follow‐up was 1447 (959) days for the SGLT‐2i group and 1459 (1000) days for DPP‐4i (Table S4). In the cohort without overweight/obesity, mean follow‐up was 886 (749) days for the SGLT‐2i group and 953 (864) days for the DPP‐4i group.

**FIGURE 3:**
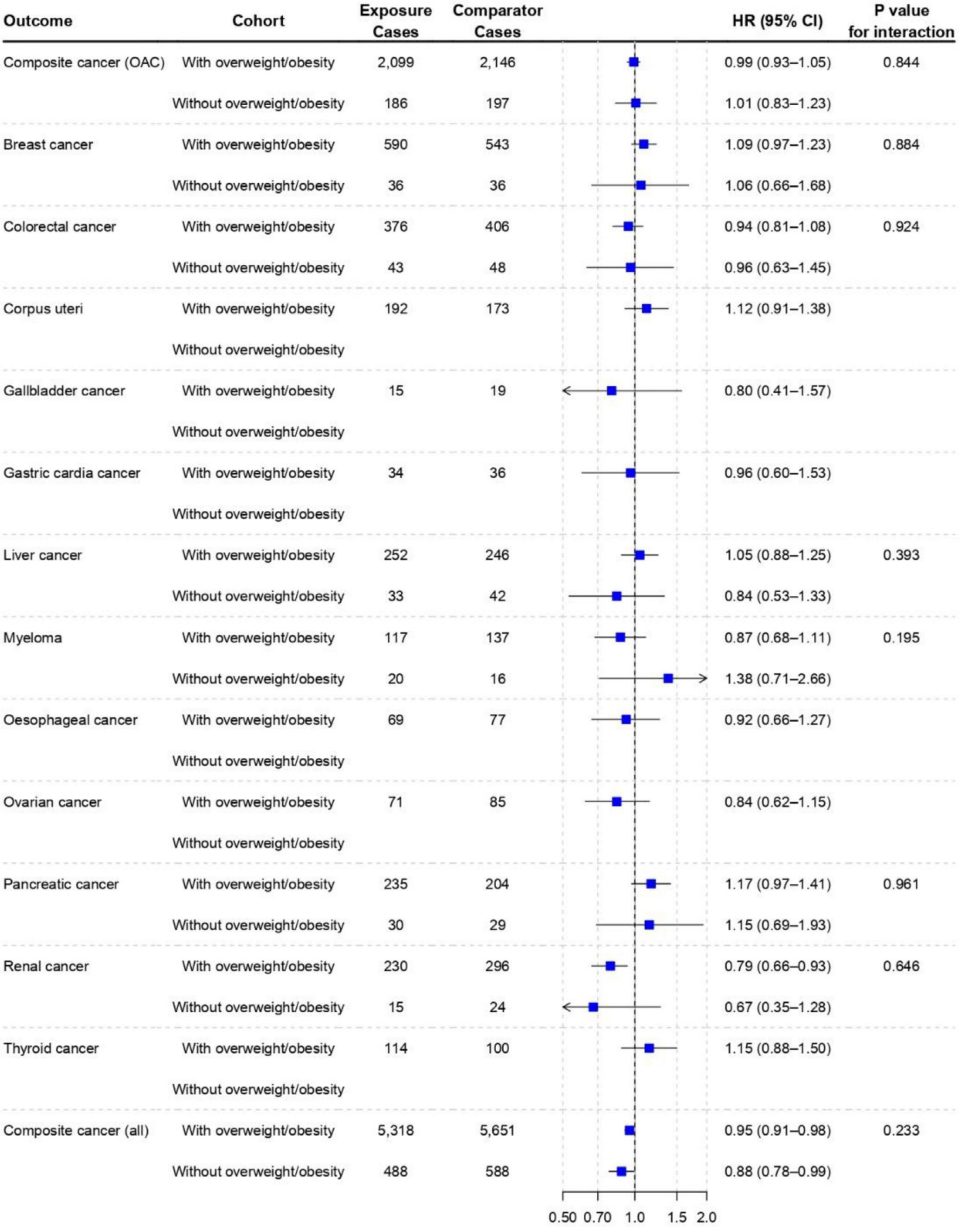
Hazard ratio of obesity‐associated cancers in individuals with type 2 diabetes with and without overweight/obesity, treated with SGLT‐2i as compared with DPP‐4i. Individuals per cohort: with overweight/obesity, 73 970; without overweight/obesity: 10 316. For several outcomes, hazard ratios could not be estimated in the cohort without overweight/obesity due to an insufficient number of events. HR: harzard ratio; CI: confidence interval; OAC: Obesity‐associated cancer.

Figure 3 shows the difference in the rate of (i) composite OAC, (ii) individual OAC types, (iii) composite all‐cancer with SGLT‐2i versus DPP‐4i in individuals with or without overweight/obesity. For composite OACs, SGLT‐2i were not associated with differences in rates compared with DPP4‐i (HR 0.99; 95% CI, 0.93–1.05) among individuals with overweight/obesity. Similarly, in individuals without overweight/obesity, there was no association between SGLT‐2i and composite OACs versus DPP‐4i (1.01; 0.83–1.23). Hazard ratios comparing SGLT‐2i versus DPP‐4i for composite OACs were not different among individuals with and without overweight/obesity (*p*_interaction_ = 0.844).

For individual OAC outcomes, SGLT‐2i were associated with lower rates of renal cancer (0.79; 0.67–0.93) versus DPP‐4i in individuals with overweight/obesity. Among individuals without overweight/obesity, no differences were observed in the rates of individual OACs between the treatment groups. No interactions were observed between SGLT‐2i and overweight/obesity status on rate of any individual OACs (*p*_interaction_ >0.05).

For the all‐cancer composite outcome, SGLT‐2i were associated with a statistically significant but modest reduction in rates compared with DPP4‐i (HR 0.95; 95% CI 0.91–0.98) among individuals with overweight/obesity. A similar association was also observed among individuals without overweight/obesity (0.88; 0.78–0.99).

### 3.4 Individual SGLT‐2i

Among individuals with T2D, 12 742 matched pairs were identified for canagliflozin versus DPP‐4i; 41 783 for dapagliflozin, 73 488 for empagliflozin, and 1056 for ertugliflozin. In all pairs, cohorts were generally well matched across characteristics (Tables S6, S7), however, there was some variation in characteristics between different pairs. There was also variation in duration of follow up across individual SGLT‐2i matched pairs, however within pairs the follow up was similar across exposed/unexposed cohorts (Table S4).

No individual SGLT‐2i was associated with differences in composite OAC or composite all‐cancer rates compared with DPP‐4i in the overall T2D population. Detailed results are presented in Figure S2.

### 3.5 Sensitivity analyses

Baseline characteristics before and after matching and follow‐up time for both sensitivity analyses are presented in the Data S2. After matching, in the first sensitivity analysis excluding individuals with GLP‐1 RA or tirzepatide use following SGLT‐2i/DPP‐4i initiation there were 62 362 matched pairs, while in the second sensitivity analysis restricted to individuals with repeated SGLT‐2i or DPP‐4i use at least 6 months after initial initiation there were 44 887 matched pairs.

In the first sensitivity analysis excluding individuals with GLP‐1 RA or tirzepatide use following SGLT‐2i/DPP‐4i initiation, rates of composite OAC remained not different across SGLT‐2i and DPP‐4i cohorts (HR 0.99; 95% CI 0.92–1.06; Figure S3). Rates of renal cancer remained lower in the SGLT‐2i cohort (0.68; 0.54–0.85), whereas the previously observed higher rate of thyroid cancer was no longer evident (1.17; 0.82–1.68). Additionally, the all‐cancer composite outcome was also no longer lower in the SGLT‐2i versus DPP‐4i cohort (0.97; 0.93–1.01).

In the second sensitivity analysis restricted to individuals with repeated SGLT‐2i or DPP‐4i use at least 6 months after initial initiation (Figure S4), the use of SGLT‐2i was associated with a lower rate of composite OAC (0.92; 0.86–0.99) versus DPP‐4i. SGLT‐2i use was also associated with a lower rate of colorectal (0.78; 0.67–0.92) and renal (0.80; 0.65–0.98) cancer, while no association was observed for thyroid cancer (1.13; 0.83–1.52). Consistent with the primary analysis, a lower rate of all‐cancer composite outcomes was also associated with SGLT‐2 use (0.93; 0.89–0.97).

## 4 DISCUSSION

This study provides a comprehensive real‐world observational comparison of SGLT‐2i and DPP‐4i in individuals with T2D, examining the rates of OACs (both as a composite outcome and by individual cancer types) as well as a composite all‐cancer outcome. For the primary outcome of composite OAC, initiation of SGLT‐2i was not associated with higher or lower rates compared with DPP‐4i among T2D individuals who were cancer‐free at baseline, either in the primary analysis or across overweight/obesity subgroups. Although a modest but statistically significant lower rate of composite OAC was observed among individuals persistent to treatment for more than 6 months (8% lower), this should be interpreted cautiously as it was not the primary analysis and may represent a chance finding. Furthermore, this analysis may have less clinical relevance as it excluded individuals who discontinue treatment, for example those who experience adverse effects.

For secondary, site‐specific outcomes, SGLT‐2i was associated with lower rates of renal cancer compared with DPP‐4i (22% lower) in the primary analysis, and this was consistently observed in the overweight/obesity subgroup and in both sensitivity analyses accounting for GLP‐1 RA confounding and in those with treatment persistence. In contrast, SGLT‐2i were associated with a higher rate of thyroid cancer compared to DPP‐4i in the primary analysis; however, this association was not observed in subgroup or sensitivity analyses and should therefore be interpreted with caution. For the secondary composite outcome of all‐cancer, SGLT‐2i were associated with a modest but statistically significant lower rate compared with DPP‐4i in the primary analysis (4% lower), among individuals with and without overweight/obesity separately, and in the sensitivity analysis of individuals persistent to treatment for >6 months. This should be interpreted cautiously as all‐cancer was not significantly different after excluding individuals that used GLP‐1 RAs after index, which could represent an important confounder. In addition, the magnitude of the observed difference was small and may be of limited clinical importance.

We also did not find evidence that effects vary significantly in those with versus without overweight/obesity; however, given varying statistical power across analyses, potential issues with multiple testing, the observational nature of the study, and that our primary aim was not to investigate these interactions, it remains unclear whether SGLT‐2i use may be more effective in preventing cancer among individuals with overweight/obesity.

The subgroup analysis across individual SGLT‐2is showed no significant associations, meaning that it remains unclear whether certain individual SGLT‐2is may be more or less effective for cancer prevention.

### 4.1 Comparison with prior studies

Evidence regarding the association between SGLT‐2i use and cancer risk remains limited and heterogeneous, particularly for OACs considered as a composite outcome. To date, most observational studies have focused on overall cancer incidence or selected site‐specific cancers, rather than evaluating composite OAC as a specific outcome. In this context, our study extends prior work by directly comparing SGLT‐2i with DPP‐4i in relation to composite OAC, individual OAC types, and composite all‐cancer outcomes in a large real‐world population with T2D. This is particularly important due to the modest WL associated with SGLT‐2i use.

Several prior observational studies have reported lower rates of overall cancer among individuals with T2D treated with SGLT‐2i compared with other glucose‐lowering therapies.^28^ For example, Chung et al. examined the association of SGLT‐2i versus DPP‐4i with new‐onset cancers, including lung, gastrointestinal, bladder, and genitourinary cancers, in Asian individuals with T2D.^29^ This study identified a significantly lower rate of all‐cancer (HR 0.70; 95% CI 0.59–0.84) and breast cancer (HR 0.51; 95% CI 0.32–0.80) among SGLT‐2i users.^29^ Similarly, Suzuki et al. conducted a retrospective study in a Japanese cohort with T2D and reported lower rates of all‐cause composite cancer (HR 0.80; 95% CI 0.70–0.91) and colorectal cancer (HR 0.71; 95% CI 0.50–0.998) with SGLT‐2i compared with DPP‐4i.^30^

In contrast, in our US‐based analysis, there was only a marginally lower rate of composite all‐cancer across cohorts which may have limited clinical meaning (4% lower). Differences in population characteristics including racial and ethnic composition (58% White, 20% Black, and 10% Asian), as well as geographic setting, comparator definitions, and confounder adjustment strategies may explain variation in effect sizes across studies. Importantly, our study excluded individuals with prior exposure to GLP‐1 RA or tirzepatide, whereas Chung et al. as well as Suzuki et al. did not apply this exclusion; therefore, observed effects in their study may reflect confounding from the GLP‐1‐based therapies rather than SGLT‐2i alone. Recent meta‐analyses of randomised controlled trials have not demonstrated significant associations between SGLT‐2i use and overall cancer incidence, despite heterogeneity across individual agents and cancer sites.^31^, ^32^

Prior studies also provide context for interpreting our secondary outcomes of site‐specific, individual OACs. Chiu et al. reported a significantly lower risk of renal cancer among SGLT‐2i users compared with non‐users in a large Taiwanese cohort (HR 0.67; 95% CI 0.55–0.81), which is consistent with our observations in the overall population and among individuals with overweight/obesity.^33^

A retrospective study conducted in a US‐based population found that SGLT‐2i use was associated with a lower rate of colorectal cancer compared with DPP‐4i use (HR 0.63; 95% CI 0.45–0.88),^34^ which is directionally consistent with our findings, albeit a much larger effect size and statistical difference as compared to the present study (HR 0.92; 95% CI 0.81–1.04). We also observed a significantly lower rate of colorectal cancer in those persistent to treatment, however, as this was not observed within the primary analysis or our primary outcome, it should be interpreted with caution. Another study evaluating breast cancer risk reported no significant difference between SGLT‐2i and DPP‐4i in a T2D population without BMI inclusion criteria.^35^ Similarly, we did not observe a statistically significant association between SGLT‐2i use and breast cancer in the overall population.

It is important to note that although we observed a significantly higher rate of thyroid cancer associated with SGLT‐2i use in the overall population, this finding has not been previously reported. Given this was a secondary outcome and that preclinical mechanistic studies have suggested a potential inhibitory effect of the SGLT‐2i canagliflozin on thyroid cancer growth, the observed association in the primary analysis should be interpreted cautiously and investigated in other populations and datasets.^36^

### 4.2 Mechanisms related to anti‐cancer effects of SGLT‐2i in renal cancer

A wide range of potentially anti‐cancer mechanisms associated with SGLT‐2i have been described previously in preclinical studies using in vitro or in vivo models. In the present analysis, for almost all outcomes investigated, these effects did not translate to real‐world findings. We did however observe a lower rate of renal cancer in association with SGLT‐2i use, consistent with pre‐clinical data suggesting potential organ‐specific anti‐cancer effects beyond WL‐related mechanisms.^19^, ^20^, ^37^ Nagata et al. demonstrated that SGLT‐2i reduce human renal cancer cell viability, colony formation, and migration, as well as decreasing glucose uptake, and also confirmed the expression of SGLT‐2 in these cells, supporting a direct antitumour effect mediated through SGLT‐2 inhibition.^38^ This study corroborates earlier preclinical evidence demonstrating that SGLT‐2i suppress renal cancer cell growth both in vitro and in vivo.^20^, ^39^

Overall, studies suggest that potential anticancer mechanisms of SGLT‐2i may be multifactorial and include suppression of glucose uptake, inhibition of the Wnt/β‐catenin pathway, reduced angiogenesis and cell adhesion, impaired DNA and RNA synthesis, and activation of the AMPK pathway.^40^ These mechanisms are especially relevant in the context of T2D, where hyperglycaemia, hyperinsulinemia, and systemic inflammation contribute to cancer risk.

### 4.3 Strengths and limitations

The primary strength of our study lies in the use of a large, real‐world global dataset, providing a more representative assessment of the association between SGLT‐2i and OAC outcomes. Unlike prior studies which have been limited by smaller sample sizes or geographic specificity, our analysis included a large diverse population. A further strength was the use of propensity score matching across a range of confounders, including BMI and HbA1c values, which is likely to minimise potential confounding. Additionally, individuals who developed cancer within the first 6 months after initiation of SGLT‐2i or DPP‐4i were excluded, as were those with prior exposure to tirzepatide or GLP‐1 RA before SGLT‐2i. These exclusions strengthen causal inference by reducing reverse causation and confounding, thereby improving the interpretability of the observed associations with SGLT‐2i use.

However, several limitations should be acknowledged. First, the retrospective design introduces inherent biases, including from missing data and potential residual confounding. Although we adjusted for proxy measures of lifestyle and social factors (such as nicotine and alcohol dependence and diagnoses related to socioeconomic and psychosocial circumstances), direct measures of smoking, physical activity, diet, and other lifestyle behaviours were not available. Residual confounding from unmeasured lifestyle and socioeconomic factors may therefore have influenced the observed HRs, potentially attenuating or exaggerating true associations. Furthermore, the dataset lacked information on the exact doses, cumulative duration, and adherence to SGLT‐2i or DPP‐4i, as well as diabetes duration. Treatment persistence and exposure duration may differ across drug classes and individual agents, potentially influencing cancer risk estimates. Although a sensitivity analysis restricted to individuals with evidence of continued use for more than 6 months was conducted to address early discontinuation, this approach does not ensure long‐term adherence or fully capture cumulative exposure. Previous real‐world studies have reported moderate persistence for both SGLT‐2 and DPP‐4 inhibitors, with approximately 50%–70% of patients remaining on therapy at 1 year, and such variability may contribute to exposure misclassification in observational analyses.^41^, ^42^ We also used clinical codes, rather than definitive measures to define cancer, which may be less accurate. Furthermore, we did not adjust our threshold of statistical significance to allow for multiple testing; therefore, findings from site‐specific cancer analyses and secondary analyses should be interpreted cautiously given the potential for type 1 error. Lastly, the data from TriNetX originates primarily from the United States. Although the cohort included individuals from diverse racial and ethnic backgrounds, the findings may not be generalisable to populations outside the United States or to healthcare systems with different prescribing patterns and cancer surveillance practices.

## 5 CONCLUSION

In this large real‐world study of individuals with T2D, initiation of SGLT‐2i was not associated with a higher or lower rate of composite OACs. For secondary outcomes, a modest but statistically significant lower rate of composite all‐cancer was associated with SGLT‐2i compared with DPP‐4i. Lower rates of renal cancer, but higher rates of thyroid cancer were observed in the SGLT‐2i group, which warrant further investigation.

## CONFLICT OF INTEREST STATEMENT

Dimitris Papamargaritis has received honoraria for lectures from Novo Nordisk, Eli Lilly, Boehringer Ingelheim, and Johnson & Johnson; consultancy fees from Regeneron; and research grants from Novo Nordisk, the Novo Nordisk UK Research Foundation, the Academy of Medical Sciences/Diabetes UK, and the National Institute for Health and Care Research (NIHR). Melanie J. Davies has acted as a consultant/advisor and speaker for Eli Lilly, Novo Nordisk, and Sanofi, has attended advisory boards for AbbVie, Amgen, AstraZeneca, Biomea Fusion, Carmot/Roche, Daewoong Pharmaceutical, Sanofi, Zealand Pharma, Regeneron, GSK, and EktaH, and as a speaker for AstraZeneca, Boehringer Ingelheim and Zuellig Pharma. She has received grants from AstraZeneca, Boehringer Ingelheim and Novo Nordisk. Francesco Zaccardi has received consulting fees from Servier, Menarini, and Daiichi Sankyo. Thomas Yates has received investigator‐initiated funding from Astra Zeneca, has acted as a consultant for Regeneron, and has received contracted research funding from the Reinsurance Group of America. Kamlesh Khunti has acted as a consultant, speaker or received grants for investigator‐initiated studies for Abbott, Astra Zeneca, Bayer, Novo Nordisk, Sanofi‐Aventis, Servier, Lilly and Merck Sharp & Dohme, Boehringer Ingelheim, Oramed Pharmaceuticals, Pfizer, Roche, Daiichi‐Sankyo, Applied Therapeutics, Embecta and Nestle Health Science.

## Supporting information


**Data S1** Supporting information.

**Data S2** Supporting information.

## Data Availability

The data that support the findings of this study are available from TriNetX. Restrictions apply to the availability of these data, which were used under license for this study. Data are available from the authors with the permission of TriNetX.
