# Supplementary material for: Sodium‐glucose co‐transporter 2 inhibitors and obesity‐associated cancers in people with type 2 diabetes: A real‐world observational study
Source: Diabetes Obes Metab. 2026 Feb 26;28(5):3807–18. doi: 10.1111/dom.70562 (PMC13071211; doi:10.1111/dom.70562)
Supplement: Supplementary file 1 — Data S1 Supporting information. [file DOM-28-3807-s002.docx]

SUPPLEMENTARY APPENDIX

**SODIUM-GLUCOSE CO-TRANSPORTER-2 INHIBITORS AND OBESITY-ASSOCIATED CANCERS IN PEOPLE WITH TYPE 2 DIABETES: A REAL-WORLD OBSERVATIONAL STUDY**

Contents

[**Supplementary Table S1:** Clinical codes utilised to define outcomes 3](#_Toc222493992)

[**Supplementary Table S2:** Clinical codes used for diagnoses in propensity score matching and study inclusion/exclusion criteria 4](#_Toc222493993)

[**Supplementary Table S3:** Baseline characteristics of individuals identified in the primary analysis before propensity score matching. 6](#_Toc222493994)

[**Supplementary Table S4:** Follow up time 8](#_Toc222493995)

[**Supplementary Table S5:** Baseline characteristics of individuals identified in individuals with obesity and individuals without obesity cohorts before and after propensity score matching. 9](#_Toc222493996)

[**Supplementary Table S6:** Individual SGLT-2is baseline characteristics before propensity score matching 13](#_Toc222493997)

[**Supplementary Table S7:** Individual SGLT-2is baseline characteristics after propensity score matching. 17](#_Toc222493998)

[**Supplementary Figure S1:** Kaplan–Meier curves for cancer outcomes by treatment group (SGLT-2i vs DPP-4i) over follow-up. 20](#_Toc222493999)

[**Supplementary Figure S2:** Hazard ratio of obesity-associated cancers in individuals with type 2 diabetes treated with canagliflozin, dapagliflozin, empagliflozin or ertugliflozin, as compared with DPP-4i. 21](#_Toc222494000)

[**Supplementary Figure S3:** Hazard ratio of sensitivity analysis excluding individuals with GLP-1 RA or tirzepatide use following SGLT-2i/DPP-4i initiation. 22](#_Toc222494001)

[**Supplementary Figure S4:** Hazard ratio of sensitivity analysis restricted to individuals with repeated SGLT-2i or DPP-4i use at least six months after initial initiation 23](#_Toc222494002)

[**RECORD checklist** 24](#_Toc222494003)

# **Supplementary Table S1:** Clinical codes utilised to define outcomes

| **Outcome** | **ICD-10-CM code** | **Code name** |
| --- | --- | --- |
| **Composite cancer all** | C14 | Malignant neoplasms of lip, oral cavity and pharynx |
|  | C26 | Malignant neoplasms of digestive organs |
|  | C39 | Malignant neoplasms of respiratory and intrathoracic organs |
|  | C41 | Malignant neoplasms of bone and articular cartilage |
|  | C44 | Melanoma and other malignant neoplasms of skin |
|  | C49 | Malignant neoplasms of mesothelial and soft tissue |
|  | C58 | Malignant neoplasms of female genital organs |
|  | C63 | Malignant neoplasms of male genital organs |
|  | C68 | Malignant neoplasms of urinary tract |
|  | C72 | Malignant neoplasms of eye, brain and other parts of central nervous system |
|  | C75 | Malignant neoplasms of thyroid and other endocrine glands |
|  | C80 | Malignant neoplasms of ill-defined, other secondary and unspecified sites |
|  | C7A | Malignant neuroendocrine tumors (C7A) |
|  | C7B | Secondary neuroendocrine tumors (C7B) |
|  | C96 | Malignant neoplasms of lymphoid, hematopoietic and related tissue |
| **Breast cancer** | C50 | Malignant neoplasm of breast |
| **Colorectal cancer** | C18 | Malignant neoplasm of colon |
|  | C19 | Malignant neoplasm of rectosigmoid junction |
|  | C20 | Malignant neoplasm of rectum |
| **Corpus uteri** | C54 | Malignant neoplasm of corpus uteri |
| **Gallbladder cancer** | C23 | Malignant neoplasm of gallbladder |
| **Gastric cardia cancer** | C16.0 | Malignant neoplasm of cardia |
| **Renal cancer** | C64 | Malignant neoplasm of kidney, except renal pelvis |
| **Liver cancer** | C22 | Malignant neoplasm of liver and intrahepatic bile ducts |
| **Multiple Myeloma** | C90 | Multiple myeloma and malignant plasma cell neoplasm |
| **Oesophageal cancer** | C15 | Malignant neoplasm of esophagus |
| **Ovarian cancer** | C56 | Malignant neoplasm of ovary |
| **Pancreatic cancer** | C25 | Malignant neoplasm of pancreas |
| **Thyroid cancer** | C73 | Malignant neoplasm of thyroid gland |

**ICD-10-CM:** International Classification of Diseases Clinical Modification (10^th^ revision)

# **Supplementary Table S2:** Clinical codes used for diagnoses in propensity score matching and study inclusion/exclusion criteria

| **Diagnoses in propensity score matching** | | |
| --- | --- | --- |
| **ICD-10-CM code** | | **Code name** |
| E11.2 | | Type 2 diabetes mellitus with kidney complications |
| E11.3 | | Type 2 diabetes mellitus with ophthalmic complications |
| E11.4 | | Type 2 diabetes mellitus with neurological complications |
| F10 | | Alcohol related disorders |
| Z87.891 | | Personal history of nicotine dependence |
| F17 | | Nicotine dependence |
| F10.2 | | Alcohol dependence |
| I10-I1A | | Hypertensive diseases |
| I20-I25 | | Ischemic heart diseases |
| I60-I69 | | Cerebrovascular diseases |
| I70 | | Atherosclerosis |
| I73.9 | | Peripheral vascular disease, unspecified |
| Z55- Z65 | | Persons with potential health hazards related to socioeconomic and psychosocial circumstances |
| **Study inclusion and exclusion criteria** | | |
|  | **Clinical code** | **Code name** |
| SGLT-2i | A10BK | Sodium-glucose co-transporter 2 (SGLT2) inhibitors |
|  | 1373458 | Canagliflozin |
|  | 1545653 | Empagliflozin |
|  | 1488564 | Dapagliflozin |
| DPP-4i | A10BH | Dipeptidyl peptidase 4 (DPP-4) inhibitors |
| Diabetes status | E11 | Type 2 diabetes mellitus |
|  | E10 | Type 1 diabetes mellitus |
| Metabolic measures | 9083 | BMI |
|  | 9037 | HBA1c |
| Bariatric surgery | 0D160ZA | Bypass stomach to jejunum, open approach |
|  | 0D1607A | Bypass stomach to jejunum with autologous tissue substitute, open approach |
|  | 43775 | Laparoscopy, surgical, gastric restrictive procedure; longitudinal gastrectomy (i.e, sleeve gastronomy) |
|  | 1014146 | Gastric restrictive procedure, with gastric bypass for morbid obesity |
|  | 427980007 | Sleeve gastrectomy with duodenal switch (deprecated 2004) |
|  | 427074001 | Laparoscopic sleeve gastrectomy |
|  | 43770 | Laparoscopy, surgical, gastric restrictive procesure; placement of adjustable gastric restrictive device (eg, gastric band and subcutaneous port components) |
|  | 0D160KA | Bypass stomach to jejunum with nonautologous tissue substitute, open approach |
|  | 426738005 | Duodenal switch |
|  | 0D160JA | Bypass stomach to jejunum with synthetic substitute, open approach |
|  | 0D168ZA | Bypass stomach to jejunum, via natural or artificial opening endoscopic |
|  | 43845 | Gastric restrictive procedure with partial gastrectomy, pylorus-preserving duodenoileostomy and ileoilestomy (50 to 100 cm common channel) to limit absorption (biliopancreatic diversion with duodenal switch) |
|  | 0D168JA | Bypass stomach to jejunum with synthetic substitute, via natural or artificial opening endoscopic |
|  | 0D16887A | Bypass stomach to jejunum with autologous tissue substitute, via natural or artificial opening endoscopic |
|  | 0D168KA | Bypass stomach to jejunum with nonautologous tissue substitute, via natural or artificial opening endoscopic |
|  | 30803004 | Printen and mason operation, high gastric bypass |
|  | 1007403 | Gastric restrictive procedure, without gastric bypass, for morbid obesity |
|  | 43644 | Laparoscopy, surgical, gastric restrictive procedure; with gastric bypass and Roux-en-Y gastroenterostomy (roux limb 150 cm or less) |
|  | 43842 | Gastric restrictive procedure, without gastric bypass, for morbid obesity; vertical-blended gastroplasty |
|  | 43846 | Gastric restrictive procedure, with gastric bypass for morbid obesity; with short limb (150 cm or less) Roux-en-Y gastroenterostomy |
|  | 43843 | Gastric restrictive procedure, without gastric bypass, for morbid obesity; other than vertical-banded gastroplasty |
|  | 43847 | Gastric restrictive procedure, with gastric bypass for morbid obesity; with small intestine reconstruction to limit absorption |
|  | 0DV64CZ | Restriction of stomach with extraluminal device, percutaneous endoscopic approach |
|  | 173747005 | Roux-en-Y gastrojejunostomy |

**ICD-10-CM:** International Classification of Diseases Clinical Modification (10^th^ revision).

**Supplementary Table S3:** Baseline characteristics of individuals identified in the primary analysis before propensity score matching.

|  | | **SGLT-2i vs DPP-4i** | | **SMD** |
| --- | --- | --- | --- | --- |
|  |  | **Exposure** | **Comparator (DPP-4i)** |  |
| **Number** | | 199,128 | 159,564 | - |
| **Age at Index** | | 62.6 (12.7) | 62.7 (13.4) | .004 |
| **Sex** | **Female** | 83033 (41.7) | 80734 (50.6) | .179 |
|  | **Male** | 115972 (58.2) | 78765 (49.4) | .179 |
|  | **Unknown sex** | 123 (0.1) | 65 (0.0) | .009 |
| **Race** | **American Indian or Alaska Native** | 1371 (0.7) | 785 (0.5) | .026 |
|  | **Asian** | 14392 (7.2) | 19223 (12.1) | .164 |
|  | **Black or African American** | 40688 (20.4) | 33226 (20.8) | .010 |
|  | **Native Hawaiian or Other Pacific Islander** | 1992 (1) | 2070 (1.3) | .028 |
|  | **White** | 123032 (61.8) | 89763 (56.3) | .113 |
|  | **Other Race** | 8121 (4.1) | 5172 (3.2) | .045 |
|  | **Unknown Race** | 9532 (4.8) | 9325 (5.8) | .047 |
| **Ethnicity** | **Hispanic or Latino** | 16899 (8.5) | 11692 (7.3) | .042 |
|  | **Not Hispanic or Latino** | 145332 (73.0) | 122987 (77.1) | .095 |
|  | **Unknown Ethnicity** | 36897 (18.5) | 24885 (15.6) | .078 |
| **BMI** | | 32.7 (7.6) | 32.1 (7.7) | .072 |
| **BMI categories** | **<25 kg/m2** | 33801 (17.0) | 31961 (20.0) | .079 |
|  | **25-30 kg/m2** | 71900 (36.1) | 57954 (36.3) | .004 |
|  | **30-35 kg/m2** | 74251 (37.3) | 55810 (35.0) | .048 |
|  | **35-40 kg/m2** | 48851 (24.5) | 35832 (22.5) | .049 |
|  | **40-45 kg/m2** | 26082 (13.1) | 19371 (12.1) | .029 |
|  | **45-50 kg/m2** | 12513 (6.3) | 9187 (5.8) | .022 |
|  | **50-55 kg/m2** | 5943 (3.0) | 4174 (2.6) | .022 |
|  | **>55 kg/m2** | 3838 (1.9) | 2775 (1.7) | .014 |
| **HbA1c** | | 8.0 (1.9) | 8.2 (1.9) | .097 |
| **HbA1c categories** | **<6.5 %** | 57084 (28.7) | 38374 (24.1) | .105 |
|  | **6.5-7.5 %** | 83058 (41.7) | 69072 (43.3) | .032 |
|  | **7.5-8.5 %** | 61087 (30.7) | 55480 (34.7) | .087 |
|  | **8.5-9.5 %** | 36198 (18.2) | 33339 (20.9) | .069 |
|  | **9.5-10.5 %** | 21625 (10.9) | 19636 (12.3) | .045 |
|  | **10.5-11.5 %** | 13968 (7.0) | 12474 (7.8) | .031 |
|  | **>11.5 %** | 17813 (9.0) | 15344 (9.6) | .023 |
| **Past medical history** | **Hypertensive diseases** | 160713 (80.7) | 122960 (77.1) | .089 |
|  | **Ischemic heart diseases** | 66490 (33.4) | 35198 (22.1) | .255 |
|  | **Type 2 diabetes mellitus with kidney complications** | 43636 (21.9) | 27710 (17.4) | .115 |
|  | **Type 2 diabetes mellitus with neurological complications** | 29258 (14.7) | 20010 (12.5) | .063 |
|  | **Nicotine dependence** | 27120 (13.6) | 15315 (9.6) | .126 |
|  | **Alcohol dependence** | 2646 (1.3) | 1444 (0.9) | .040 |
|  | **Cerebrovascular diseases** | 21614 (10.9) | 16146 (10.1) | .024 |
|  | **Type 2 diabetes mellitus with ophthalmic complications** | 10274 (5.2) | 7795 (4.9) | .013 |
|  | **Atherosclerosis** | 11824 (5.9) | 7123 (4.5) | .066 |
|  | **Personal history of nicotine dependence** | 36184 (18.2) | 18461 (11.6) | .186 |
|  | **Peripheral vascular disease, unspecified** | 11720 (5.9) | 7322 (4.6) | .058 |
|  | **Alcohol related disorders** | 6822 (3.4) | 3289 (2.1) | .084 |
| **Medications** | **Metformin** | 101876 (51.2) | 97043 (60.8) | .195 |
|  | **Insulin** | 77925 (39.1) | 62572 (39.2) | .002 |
|  | **Antihypertensives** | 31081 (15.6) | 25159 (15.8) | .004 |
|  | **Sulfonylureas** | 34718 (17.4) | 49274 (30.9) | .318 |
|  | **Lipid modifying agents** | 137022 (68.8) | 103349 (64.8) | .086 |
|  | **Bupropion** | 6419 (3.2) | 4405 (2.8) | .027 |
|  | **Topiramate** | 2026 (1.0) | 1584 (1.0) | .002 |
|  | **Phentermine** | 450 (0.2) | 395 (0.3) | .004 |
|  | **Naltrexone** | 561 (0.3) | 234 (0.2) | .029 |
|  | **Orlistat** | 22 (0.0) | 30 (0.0) | .006 |
| **Persons with potential health hazards related to socioeconomic and psychosocial circumstances** | | 8518 (4.3) | 3740 (2.3) | .108 |

Data are shown as mean (SD) for continuous variables and number (%) for categorical variables. **BMI:** Body mass index. Orlistat, bupropion, naltrexone, and phentermine, topiramate were not included as propensity score matching covariates but are presented for additional information.

**Supplementary Table S4:** Follow up time

**3.1 Primary analysis**

|  |  | Cohort | Mean Follow-up (Days) | Standard Deviation | Median Follow-up (Days) | Interquartile Range |
| --- | --- | --- | --- | --- | --- | --- |
|  |  | SGLT-2i | 1343 | 944.6 | 1189 | 1348 |
|  |  | DPP-4i | 1366.7 | 993.7 | 1232 | 1468 |

**3.2 Individuals with overweight/obesity**

|  |  | Cohort | Mean Follow-up (Days) | Standard Deviation | Median Follow-up (Days) | Interquartile Range |
| --- | --- | --- | --- | --- | --- | --- |
|  |  | SGLT-2i | 1446.6 | 958.7 | 1315 | 1396 |
|  |  | DPP-4i | 1459.3 | 999.5 | 1351 | 1468 |

**3.3 Individuals without overweight/obesity**

|  |  | Cohort | Mean Follow-up (Days) | Standard Deviation | Median Follow-up (Days) | Interquartile Range |
| --- | --- | --- | --- | --- | --- | --- |
|  |  | SGLT-2i | 886.4 | 748.9 | 737 | 987.5 |
|  |  | DPP-4i | 953.2 | 864.4 | 735 | 1168 |

**3.4 Individual SGLT-2i**

| **Analysis** | **Cohort** | **Mean follow-up time (days)** | **Standard deviation** | **Median follow-up time (days)** | **Interquartile range** |
| --- | --- | --- | --- | --- | --- |
| Canagliflozin vs. DPP-4i | Canagliflozin | 2172.0 | 1116.1 | 2191 | 1762 |
|  | DPP-4i | 2142.6 | 1388.1 | 2011 | 2024 |
| Dapagliflozin vs. DPP-4i | Dapagliflozin | 920.8 | 757.3 | 783 | 905 |
|  | DPP-4i | 971.4 | 847.1 | 782 | 1110 |
| Empagliflozin vs. DPP-4i | Empagliflozin | 1154.1 | 787.3 | 1057 | 1159.5 |
|  | DPP-4i | 1182.9 | 868.2 | 1071 | 1296 |
| Ertugliflozin vs DPP-4i | Ertugliflozin | 1219.1 | 717.6 | 1198 | 1079.5 |
|  | DPP-4i | 1199.3 | 926.8 | 1074.5 | 1378.5 |

# **Supplementary Table S5:** Baseline characteristics of individuals identified in individuals with obesity and individuals without obesity cohorts before and after propensity score matching.

|  | | **Before** | | | | | | **After** | | | | | |
| --- | --- | --- | --- | --- | --- | --- | --- | --- | --- | --- | --- | --- | --- |
|  | | **Individuals with overweight/obesity**  **SGLT-2i vs DPP-4i** | | **SMD** | **Individuals without overweight/obesity**  **SGLT-2i vs DPP-4i** | | **SMD** | **Individuals with overweight/obesity**  **SGLT-2i vs DPP-4i** | | **SMD** | **Individuals without overweight/obesity**  **SGLT-2i vs DPP-4i** | | **SMD** |
|  |  | **Exposure** | **Comparator (DPP-4i)** |  | **Exposure** | **Comparator (DPP-4i)** |  | **Exposure** | **Comparator (DPP-4i)** |  | **Exposure** | **Comparator (DPP-4i)** |  |
| **Number** | | 164,717 | 127,174 | - | 18,211 | 19,165 |  | 73,970 | 73,970 | - | 10,316 | 10,316 | - |
| **Age at Index** | | 61.6 (12.6) | 61.2 (13.0) | .027 | 68.2 (12.3) | 69.2 (13.1) | .078 | 61.4 (12.2) | 61.5 (13.8) | .007 | 69.4 (11.8) | 69.7 (14.1) | .023 |
| **Sex** | **Female** | 68431 (41.6) | 64213 (50.5) | .180 | 8092 (44.4) | 10115 (52.8) | .168 | 35380 (47.8) | 35392 (47.9) | .000 | 5151 (49.9) | 5180 (50.2) | .006 |
|  | **Male** | 96185 (58.4) | 62911 (49.5) | .180 | 10107 (55.5) | 9040 (47.2) | .167 | 38546 (52.1) | 38535 (52.1) | .000 | 5161 (50.0) | 5129 (49.7) | .006 |
|  | **Unknown sex** | 101 (0.1) | 50 (0.0) | .010 | 12 (0.1) | 10 (0.1) | .006 | 44 (0.1) | 43 (0.1) | .001 | 10 (0.1) | 10 (0.1) | - |
| **Race** | **American Indian or Alaska Native** | 1102 (0.7) | 627 (0.5) | .023 | 139 (0.8) | 93 (0.5) | .035 | 401 (0.5) | 422 (0.6) | .004 | 72 (0.7) | 68 (0.7) | .005 |
|  | **Asian** | 8754 (5.3) | 10221 (8.0) | .109 | 3988 (21.9) | 6722 (35.1) | .295 | 5209 (7.0) | 5108 (6.9) | .005 | 2829 (27.4) | 2832 (27.5) | .001 |
|  | **Black or African American** | 33744 (20.5) | 27487 (21.6) | .028 | 3440 (18.9) | 2849 (14.9) | .108 | 15395 (20.8) | 15413 (20.8) | .001 | 1793 (17.4) | 1760 (17.1) | .008 |
|  | **Native Hawaiian or Other Pacific Islander** | 1729 (1.1) | 1675 (1.3) | .025 | 128 (0.7) | 182 (1.0) | .027 | 813 (1.1) | 818 (1.1) | .001 | 83 (0.8) | 77 (0.8) | .007 |
|  | **White** | 105458 (64.0) | 76678 (60.3) | .077 | 8325 (45.7) | 6721 (35.1) | .218 | 45400 (61.4) | 45517 (61.5) | .003 | 4132 (40.1) | 4152 (40.3) | .004 |
|  | **Other Race** | 6453 (3.9) | 3873 (3.1) | .0476 | 947 (5.2) | 751 (3.9) | .061 | 2738 (3.7) | 2705 (3.7) | .002 | 511 (5.0) | 485 (4.7) | .012 |
|  | **Unknown Race** | 7477 (4.5) | 6613 (5.2) | .031 | 1244 (6.8) | 1847 (9.6) | .102 | 4014 (5.4) | 3987 (5.4) | .002 | 896 (8.7) | 942 (9.1) | .016 |
| **Ethnicity** | **Hispanic or Latino** | 13641 (8.3) | 9043 (7.1) | .044 | 1667 (9.2) | 1323 (6.9) | .083 | 5951 (8.1) | 5940 (8.0) | .001 | 844 (8.2) | 833 (8.1) | .004 |
|  | **Not Hispanic or Latino** | 119711 (72.7) | 98031 (77.1) | .102 | 13389 (73.5) | 14775 (77.1) | .083 | 54533 (73.7) | 54533 (73.7) | - | 7634 (74.0) | 7615 (73.8) | .004 |
|  | **Unknown Ethnicity** | 31365 (19.0) | 20100 (15.8) | .085 | 3155 (17.3) | 3067 (16.0) | .035 | 13486 (18.2) | 13497 (18.3) | .000 | 1838 (17.8) | 1868 (18.1) | .008 |
| **BMI** | | 34.51 (6.9) | 34.3 (6.8) | .028 | 22.2 (2.0) | 22.1 (2.1) | .067 | 34.4 (6.8) | 34.3 (6.8) | .014 | 22.2 (2.0) | 22.0 (2.1) | .088 |
| **BMI categories** | **<25 kg/m2** | 81 (0.1) | 51 (0.0) | .004 | 18211 (100) | 19165 (100) | - | 33 (0.1) | 33 (0.1) | - | 10316 (100) | 10316 (100) | - |
|  | **25-30 kg/m2** | 57106 (34.7) | 45804 (36.0) | .028 | 58 (0.3) | 38 (0.2) | .024 | 26368 (35.7) | 26487 (35.8) | .003 | 26 (0.3) | 26 (0.3) | - |
|  | **30-35 kg/m2** | 71842 (43.6) | 53840 (42.3) | .026 | 10 (0.1) | 10 (0.1) | .001 | 31424 (42.5) | 31407 (42.5) | .000 | 10 (0.1) | 10 (0.1) | - |
|  | **35-40 kg/m2** | 47886 (29.1) | 35159 (27.7) | .032 | 10 (0.1) | 0 (0) | .033 | 20726 (28.0) | 20691 (28.0) | .001 | 0 (0) | 0 (0) | - |
|  | **40-45 kg/m2** | 25636 (15.6) | 19051 (15.0) | .016 | 10 (0.1) | 0 (0) | .033 | 10956 (14.8) | 11126 (15.0) | .00 | 0 (0) | 0 (0) | - |
|  | **45-50 kg/m2** | 12281 (7.5) | 9042 (7.1) | .013 | 0 (0) | 0 (0) | - | 5251 (7.1) | 5313 (7.2) | .003 | 0 (0) | 0 (0) | - |
|  | **50-55 kg/m2** | 5829 (3.5) | 4098 (3.2) | .018 | 0 (0) | 0 (0) | - | 2430 (3.3) | 2440 (3.3) | .001 | 0 (0) | 0 (0) | - |
|  | **>55 kg/m2** | 3727 (2.3) | 2668 (2.1) | .011 | 0 (0) | 0 (0) | - | 1519 (2.1) | 1572 (2.1) | .005 | 0 (0) | 0 (0) | - |
| **HbA1c** | | 8.1 (1.9) | 8.3 (1.9) | .103 | 7.9 (2.1) | 8.1 (2.1) | .077 | 8.3 (1.9) | 8.3 (1.9) | .007 | 8.0 (2.0) | 8.0 (2.1) | .022 |
| **HbA1c categories** | **<6.5 %** | 44690 (27.1) | 28304 (22.3) | .113 | 5920 (32.5) | 5428 (28.3) | .091 | 16423 (22.2) | 16774 (22.7) | .011 | 3025 (29.3) | 3002 (29.1) | .005 |
|  | **6.5-7.5 %** | 69197 (42.0) | 54878 (43.2) | .023 | 7151 (39.3) | 8193 (42.8) | .071 | 31226 (42.2) | 31124 (42.1) | .003 | 4299 (41.7) | 4275 (41.4) | .005 |
|  | **7.5-8.5 %** | 51860 (31.5) | 45475 (35.8) | .091 | 4850 (26.6) | 5772 (30.1) | .077 | 25582 (34.6) | 25493 (34.5) | .003 | 2955 (28.7) | 2923 (28.3) | .007 |
|  | **8.5-9.5 %** | 30939 (18.8) | 27572 (21.7) | .072 | 2703 (14.8) | 3249 (17.0) | .058 | 15676 (21.2) | 15573 (21.1) | .003 | 1639 (15.9) | 1618 (15.7) | .006 |
|  | **9.5-10.5 %** | 18541 (11.3) | 16290 (12.8) | .0478 | 1600 (8.8) | 1875 (9.8) | .034 | 9444 (12.8) | 9279 (12.5) | .007 | 937 (9.1) | 984 (9.5) | .016 |
|  | **10.5-11.5 %** | 11925 (7.2) | 10288 (8.1) | .032 | 1074 (5.9) | 1251 (6.5) | .026 | 5991 (8.1) | 6022 (8.1) | .002 | 652 (6.3) | 633 (6.1) | .008 |
|  | **>11.5 %** | 14468 (8.8) | 12026 (9.5) | .023 | 1831 (10.1) | 2007 (10.5) | .014 | 7240 (9.8) | 7075 (9.6) | .008 | 1046 (10.1) | 1069 (10.4) | .007 |
| **Past medical history** | **Hypertensive diseases** | 133244 (80.9) | 98863 (77.7) | .078 | 13606 (74.7) | 13314 (69.5) | .117 | 57737 (78.1) | 57945 (78.3) | .007 | 7440 (72.1) | 7439 (72.1) | .000 |
|  | **Ischemic heart diseases** | 51459 (31.2) | 25662 (20.2) | .255 | 6749 (37.1) | 4737 (24.7) | .270 | 16655 (22.5) | 16920 (22.9) | .009 | 3012 (29.2) | 3029 (29.4) | .004 |
|  | **Type 2 diabetes mellitus with kidney complications** | 34177 (20.8) | 19925 (15.7) | .132 | 4281 (23.5) | 3910 (20.4) | .075 | 13390 (18.1) | 13579 (18.4) | .007 | 2328 (22.6) | 2335 (22.6) | .002 |
|  | **Type 2 diabetes mellitus with neurological complications** | 23668 (14.4) | 15635 (12.3) | .061 | 2450 (13.5) | 2055 (10.7) | .084 | 10328 (14.0) | 10299 (13.9) | .001 | 1331 (12.9) | 1305 (12.7) | .008 |
|  | **Nicotine dependence** | 21025 (12.8) | 11976 (9.4) | .107 | 2899 (15.9) | 1630 (8.5) | .228 | 7777 (10.5) | 7860 (10.6) | .004 | 1118 (10.8) | 1113 (10.8) | .002 |
|  | **Alcohol dependence** | 1864 (1.1) | 912 (0.7) | .043 | 356 (2.0) | 250 (1.3) | .051 | 638 (0.9) | 639 (0.9) | .000 | 171 (1.7) | 151 (1.5) | .016 |
|  | **Cerebrovascular diseases** | 15609 (9.5) | 10700 (8.4) | .037 | 2735 (15.0) | 2857 (14.9) | .003 | 6397 (8.7) | 6459 (8.7) | .003 | 1512 (14.7) | 1520 (14.7) | .002 |
|  | **Type 2 diabetes mellitus with ophthalmic complications** | 7924 (4.8) | 5538 (4.4) | .022 | 1148 (6.3) | 1165 (6.1) | .009 | 3540 (4.8) | 3459 (4.7) | .005 | 652 (6.3) | 660 (6.4) | .003 |
|  | **Atherosclerosis** | 8273 (5.0) | 4535 (3.6) | .072 | 1528 (8.4) | 1209 (6.3) | .080 | 3046 (4.1) | 3092 (4.2) | .003 | 766 (7.4) | 772 (7.5) | .002 |
|  | **Personal history of nicotine dependence** | 28699 (17.4) | 14037 (11.0) | .184 | 2932 (16.1) | 1797 (9.4) | .203 | 10151 (13.7) | 10220 (13.8) | .003 | 1278 (12.4) | 1241 (12.0) | .011 |
|  | **Peripheral vascular disease, unspecified** | 8345 (5.1) | 4920 (3.9) | .058 | 1486 (8.3) | 1126 (5.9) | .090 | 3048 (4.1) | 3104 (4.2) | .004 | 680 (6.6) | 701 (6.8) | .008 |
|  | **Alcohol related disorders** | 5012 (3.0) | 2234 (1.8) | .084 | 785 (4.3) | 496 (2.6) | .094 | 1569 (2.1) | 1585 (2.1) | .001 | 326 (3.2) | 312 (3.0) | .008 |
| **Medications** | **Metformin** | 85653 (52) | 79348 (62.4) | .211 | 8902 (48.9) | 10845 (56.6) | .155 | 44117 (59.6) | 43714 (59.1) | .011 | 5515 (53.5) | 5553 (53.8) | .007 |
|  | **Insulin** | 61160 (37.1) | 45487 (35.8) | .028 | 7600 (41.7) | 8894 (46.4) | .094 | 26661 (36.0) | 26874 (36.3) | .006 | 4496 (43.6) | 4579 (44.4) | .016 |
|  | **Antihypertensives** | 23880 (14.5) | 18141 (14.3) | .007 | 2821 (15.5) | 3064 (16.0) | .014 | 10055 (13.6) | 10272 (13.9) | .009 | 1597 (15.5) | 1614 (15.7) | .005 |
|  | **Sulfonylureas** | 28961 (17.6) | 40018 (31.5) | .327 | 3122 (17.1) | 5417 (28.8) | .268 | 18403 (24.9) | 18117 (24.5) | .009 | 2204 (21.4) | 2219 (21.5) | .004 |
|  | **Lipid modifying agents** | 111510 (67.7) | 81939 (64.4) | .069 | 13037 (71.6) | 12036 (62.8) | .188 | 47821 (64.7) | 47800 (64.6) | .001 | 6936 (67.2) | 6945 (67.3) | .002 |
|  | **Bupropion** | 5642 (3.4) | 3809 (3.0) | .024 | 290 (1.6) | 250 (1.3) | .024 | 2465 (3.3) | 2313 (3.1) | .012 | 128 (1.2) | 154 (1.5) | .022 |
|  | **Topiramate** | 1809 (1.1) | 1408 (1.1) | .001 | 76 (0.4) | 69 (0.4) | .009 | 808 (1.1) | 905 (1.2) | .012 | 44 (0.4) | 42 (0.4) | .003 |
|  | **Phentermine** | 443 (0.3) | 391 (0.3) | .007 | 10 (0.1) | 0 (0) | .033 | 277 (0.4) | 202 (0.3) | .018 | 23 (0.2) | 14 (0.1) | .044 |
|  | **Naltrexone** | 445 (0.3) | 189 (0.2) | .027 | 55 (0.3) | 17 (0.1) | .048 | 183 (0.3) | 145 (0.2) | .011 | 10 (0.1) | 0 (0) | .021 |
|  | **Orlistat** | 21 (0.0) | 30 (0.0) | .008 | 0 (0) | 0 (0) | - | 13 (0.0) | 10 (0.01) | .003 | 0 (0) | 0 (0) | - |
| **Persons with potential health hazards related to socioeconomic and psychosocial circumstances** | | 6392 (3.9) | 2687 (2.1) | .104 | 888 (4.9) | 472 (2.5) | .129 | 2128 (2.9) | 2047 (2.8) | .007 | 373 (3.6) | 346 (3.4) | .014 |

Data are shown as median (SD) for continuous variables and number (%) for categorical variables. **BMI:** Body mass index. Orlistat, bupropion, naltrexone, and phentermine, topiramate were not included as propensity score matching covariates but are presented for additional information.

# **Supplementary Table S6:** Individual SGLT-2is baseline characteristics before propensity score matching

|  | | **Canagliflozin vs DPP-4i** | | | **Dapagliflozin vs DPP-4i** | | | **Empagliflozin vs DPP-4i** | | | **Ertugliflozin vs DPP-4i** | | |  |
| --- | --- | --- | --- | --- | --- | --- | --- | --- | --- | --- | --- | --- | --- | --- |
|  |  | **Exposure** | **Comparator (DPP-4i)** | **SMD** | **Exposure** | **Comparator (DPP-4i)** | **SMD** | **Exposure** | **Comparator (DPP-4i)** | **SMD** | **Exposure** | **Comparator (DPP-4i)** | **SMD** |  |
| **Number** | | 12743 | 159564 | - | 52801 | 159564 | - | 130791 | 159564 | - | 1060 | 159564 | - |  |
| **Age at Index** | | 57.9 (11.8) | 62.7 (13.4) | .379 | 63.69874 (12.8) | 62.7 (13.4) | .078 | 62.7 (12.7) | 62.7 (13.4) | .003 | 54.7 (12.3) | 62.7 (13.4) | .621 |  |
| **Sex** | **Female** | 5705 (44.8) | 80734 (50.6) | .117 | 21786 (41.3) | 80734 (50.6) | .188 | 54353 (41.6) | 80734 (50.6) | .182 | 499 (47.1) | 80734 (50.6) | .070 |  |
|  | **Male** | 7032 (55.2) | 78765 (49.4) | .117 | 30990 (58.7) | 78765 (49.4) | .188 | 76350 (58.4) | 78765 (49.4) | .182 | 558 (52.6) | 78765 (49.4) | .066 |  |
|  | **Unknown sex** | 10 (0.1) | 65 (0.0) | .015 | 25 (0.1) | 65 (0.0) | .003 | 88 (0.1) | 65 (0.0) | .011 | 10 (0.9) | 65 (0.0) | .129 |  |
| **Race** | **American Indian or Alaska Native** | 64 (0.5) | 785 (0.5) | .001 | 283 (0.5) | 785 (0.5) | .006 | 999 (0.8) | 785 (0.5) | .034 | 11 (1.0) | 785 (0.5) | .063 |  |
|  | **Asian** | 836 (6.6) | 19223 (12.1) | .190 | 4731 (9.0) | 19223 (12.1) | .101 | 8617 (6.6) | 19223 (12.1) | .189 | 102 (9.6) | 19223 (12.1) | .078 |  |
|  | **Black or African American** | 2202 (17.3) | 33226 (20.8) | .090 | 11512 (21.8) | 33226 (20.8) | .024 | 26428 (20.2) | 33226 (20.8) | .015 | 218 (20.6) | 33226 (20.8) | .006 |  |
|  | **Native Hawaiian or Other Pacific Islander** | 96 (0.8) | 2070 (1.3) | .054 | 371 (0.7) | 2070 (1.3) | .060 | 1456 (1.1) | 2070 (1.3) | .017 | 44 (4.2) | 2070 (1.3) | .176 |  |
|  | **White** | 8447 (66.3) | 89763 (56.3) | .207 | 31576 (59.8) | 89763 (56.3) | .072 | 81391 (62.2) | 89763 (56.3) | .122 | 519 (49.0) | 89763 (56.3) | .146 |  |
|  | **Other Race** | 480 (3.8) | 5172 (3.2) | .029 | 1830 (3.5) | 5172 (3.2) | .012 | 5636 (4.3) | 5172 (3.2) | .056 | 89 (8.4) | 5172 (3.2) | .222 |  |
|  | **Unknown Race** | 618 (4.9) | 9325 (5.8) | .044 | 9325 (5.8) | 9325 (5.8) | .050 | 6264 (4.8) | 9325 (5.8) | .047 | 77 (7.3) | 9325 (5.8) | .057 |  |
| **Ethnicity** | **Hispanic or Latino** | 1117 (8.8) | 11692 (7.3) | .0529 | 4060 (7.7) | 11692 (7.3) | .014 | 11402 (8.7) | 11692 (7.3) | .051 | 158 (14.9) | 11692 (7.3) | .243 |  |
|  | **Not Hispanic or Latino** | 9750 (76.5) | 122987 (77.1) | .013 | 39074 (74.0) | 122987 (77.1) | .072 | 94584 (72.3) | 122987 (77.1) | .110 | 706 (66.6) | 122987 (77.1) | .234 |  |
|  | **Unknown Ethnicity** | 1876 (14.7) | 24885 (15.6) | .024 | 9667 (18.3) | 24885 (15.6) | .072 | 24805 (19.0) | 24885 (15.6) | .089 | 196 (18.5) | 24885 (15.6) | .077 |  |
| **BMI** | | 34.5 (7.5) | 32.1 (7.7) | .311 | 32.5 (7.8) | 32.1 (7.7) | .040 | 32.6 (7.5) | 32.1 (7.7) | .062 | 33.4 (8.0) | 32.1 (7.7) | .156 |  |
| **BMI categories** | **<25 kg/m2** | 1182 (9.3) | 31961 (20.0) | .308 | 10126 (19.2) | 31961 (20.0) | .021 | 22031 (16.8) | 31961 (20.0) | .082 | 178 (16.8) | 31961 (20.0) | .084 |  |
|  | **25-30 kg/m2** | 3602 (28.3) | 57954 (36.3) | .173 | 19701 (37.3) | 57954 (36.3) | .021 | 47635 (36.4) | 57954 (36.3) | .002 | 360 (34.0) | 57954 (36.3) | .049 |  |
|  | **30-35 kg/m2** | 4728 (37.1) | 55810 (35.0) | .044 | 19472 (36.9) | 55810 (35.0) | .040 | 49020 (37.5) | 55810 (35.0) | .052 | 366 (34.5) | 55810 (35.0) | .009 |  |
|  | **35-40 kg/m2** | 3625 (28.5) | 35832 (22.5) | .138 | 12744 (24.1) | 35832 (22.5) | .040 | 31765 (24.3) | 35832 (22.5) | .043 | 260 (24.5) | 35832 (22.5) | .049 |  |
|  | **40-45 kg/m2** | 2149 (16.9) | 19371 (12.1) | .134 | 6988 (13.2) | 19371 (12.1) | .033 | 16533 (12.6) | 19371 (12.1) | .015 | 156 (14.7) | 19371 (12.1) | .076 |  |
|  | **45-50 kg/m2** | 1003 (7.9) | 9187 (5.8) | .084 | 3436 (6.5) | 9187 (5.8) | .031 | 7883 (6.0) | 9187 (5.8) | .011 | 80 (7.6) | 9187 (5.8) | .072 |  |
|  | **50-55 kg/m2** | 446 (3.5) | 4174 (2.6) | .051 | 1715 (3.3) | 4174 (2.6) | .037 | 3680 (2.8) | 4174 (2.6) | .012 | 42 (4.0) | 4174 (2.6) | .076 |  |
|  | **>55 kg/m2** | 276 (2.2) | 2775 (1.7) | .031 | 1139 (2.2) | 2775 (1.7) | .030 | 2359 (1.8) | 2775 (1.7) | .005 | 24 (2.3) | 2775 (1.7) | .037 |  |
| **HbA1c** | | 8.5 (1.9) | 8.2 (1.9) | .159 | 7.7406325 (1.9) | 8.2 (1.9) | .244 | 8.1 (1.9) | 8.2 (1.9) | .066 | 8.8 (2.2) | 8.2 (1.9) | 0.278 |  |
| **HbA1c categories** | **<6.5 %** | 2148 (16.9) | 38374 (24.1) | .179 | 18663 (35.4) | 38374 (24.1) | .249 | 35571 (27.2) | 38374 (24.1) | .072 | 208 (19.6) | 38374 (24.1) | .107 |  |
|  | **6.5-7.5 %** | 5081 (39.9) | 69072 (43.3) | .069 | 21421 (40.6) | 69072 (43.3) | .056 | 55513 (42.4) | 69072 (43.3) | .017 | 352 (33.2) | 69072 (43.3) | .209 |  |
|  | **7.5-8.5 %** | 5021 (39.4) | 55480 (34.8) | .096 | 13667 (25.9) | 55480 (34.8) | .194 | 41549 (31.8) | 55480 (34.8) | .064 | 319 (30.1) | 55480 (34.8) | .100 |  |
|  | **8.5-9.5 %** | 3282 (25.8) | 33339 (20.9) | .115 | 7726 (14.6) | 33339 (20.9) | .164 | 24603 (18.8) | 33339 (20.9) | .052 | 233 (22.0) | 33339 (20.9) | .026 |  |
|  | **9.5-10.5 %** | 2047 (16.1) | 19636 (12.3) | .108 | 4598 (8.7) | 19636 (12.3) | .118 | 14612 (11.2) | 19636 (12.3) | .035 | 170 (16.0) | 19636 (12.3) | .107 |  |
|  | **10.5-11.5 %** | 1364 (10.7) | 12474 (7.8) | .100 | 3018 (5.7) | 12474 (7.8) | .084 | 9354 (7.2) | 12474 (7.8) | .025 | 119 (11.2) | 12474 (7.8) | .116 |  |
|  | **>11.5 %** | 1369 (10.7) | 15344 (9.6) | .037 | 4024 (7.6) | 15344 (9.6) | .071 | 12083 (9.2) | 15344 (9.6) | .013 | 182 (17.2) | 15344 (9.6) | .223 |  |
| **Past medical history** | **Hypertensive diseases** | 9511 (74.6) | 122960 (77.1) | .057 | 44186 (83.7) | 122960 (77.1) | .167 | 104850 (80.2) | 122960 (77.1) | .076 | 723 (68.2) | 122960 (77.1) | .200 |  |
|  | **Ischemic heart diseases** | 2210 (17.3) | 35198 (22.1) | .119 | 21448 (40.6) | 35198 (22.1) | .408 | 42063 (32.2) | 35198 (22.1) | .229 | 149 (14.1) | 35198 (22.1) | .209 |  |
|  | **Type 2 diabetes mellitus with kidney complications** | 1491 (11.7) | 27710 (17.4) | .161 | 14318 (27.1) | 27710 (17.4) | .236 | 27262 (20.8) | 27710 (17.4) | .089 | 131 (12.4) | 27710 (17.4) | .141 |  |
|  | **Type 2 diabetes mellitus with neurological complications** | 1599 (12.6) | 20010 (12.5) | .000 | 8263 (15.7) | 20010 (12.5) | .0894 | 18976 (14.5) | 20010 (12.5) | .058 | 153 (14.4) | 20010 (12.5) | .055 |  |
|  | **Nicotine dependence** | 1227 (9.6) | 15315 (9.6) | .001 | 7778 (14.7) | 15315 (9.6) | .158 | 17668 (13.5) | 15315 (9.6) | .123 | 178 (16.8) | 15315 (9.6) | .214 |  |
|  | **Alcohol dependence** | 119 (0.9) | 1444 (0.9) | .003 | 755 (1.4) | 1444 (0.9) | .049 | 1729 (1.3) | 1444 (0.9) | .040 | 25 (2.4) | 1444 (0.9) | .115 | |
|  | **Cerebrovascular diseases** | 699 (5.5) | 16146 (10.1) | .173 | 7036 (13.3) | 16146 (10.1) | .100 | 13628 (10.4) | 16146 (10.1) | .010 | 72 (6.8) | 16146 (10.1) | .120 |  |
|  | **Type 2 diabetes mellitus with ophthalmic complications** | 615 (4.8) | 7795 (4.9) | .003 | 2523 (4.8) | 7795 (4.9) | .005 | 6992 (5.4) | 7795 (4.9) | .021 | 46 (4.3) | 7795 (4.9) | .026 |  |
|  | **Atherosclerosis** | 343 (2.7) | 7123 (4.5) | .096 | 4042 (7.7) | 7123 (4.5) | .134 | 7327 (5.6) | 7123 (4.5) | .052 | 24 (2.3) | 7123 (4.5) | .122 |  |
|  | **Personal history of nicotine dependence** | 1288 (10.1) | 18461 (11.6) | .047 | 10820 (20.5) | 18461 (11.6) | .245 | 23582 (18.0) | 18461 (11.6) | .183 | 134 (12.6) | 18461 (11.6) | .033 |  |
|  | **Peripheral vascular disease, unspecified** | 375 (2.9) | 7322 (4.6) | .087 | 3789 (7.2) | 7322 (4.6) | .110 | 7430 (5.7) | 7322 (4.6) | .049 | 28 (2.6) | 7322 (4.6) | .104 |  |
|  | **Alcohol related disorders** | 272 (2.1) | 3289 (2.1) | .005 | 2073 (3.9) | 3289 (2.1) | .110 | 4365 (3.3) | 3289 (2.1) | .079 | 44 (4.2) | 3289 (2.1) | .121 |  |
| **Medications** | **Metformin** | 8195 (64.3) | 97043 (60.8) | .072 | 22423 (42.5) | 97043 (60.8) | .374 | 69658 (53.3) | 97043 (60.8) | .153 | 730 (68.9) | 97043 (60.8) | .169 |  |
|  | **Insulin** | 4122 (32.4) | 62572 (39.2) | .144 | 24568 (46.5) | 62572 (39.2) | .148 | 48031 (36.7) | 62572 (39.2) | .051 | 418 (39.4) | 62572 (39.2) | .004 |  |
|  | **Antihypertensives** | 1062 (8.3) | 25159 (15.8) | .230 | 11836 (22.4) | 25159 (15.8) | .170 | 17774 (13.6) | 25159 (15.8) | .062 | 112 (10.6) | 25159 (15.8) | .154 |  |
|  | **Sulfonylureas** | 3651 (28.7) | 49274 (30.9) | .049 | 7678 (14.5) | 49274 (30.9) | .398 | 22791 (17.4) | 49274 (30.9) | .318 | 279 (26.32) | 49274 (30.9) | .101 |  |
|  | **Lipid modifying agents** | 7598 (59.6) | 103349 (64.8) | .106 | 37828 (71.6) | 103349 (64.8) | .148 | 89687 (68.6) | 103349 (64.8) | .081 | 645 (60.9) | 103349 (64.8) | .081 |  |
|  | **Bupropion** | 438 (3.4) | 4405 (2.8) | .039 | 1606 (3.0) | 4405 (2.8) | .017 | 4272 (3.3) | 4405 (2.8) | .030 | 38 (3.6) | 4405 (2.8) | .047 |  |
|  | **Topiramate** | 139 (1.1) | 1584 (1.0) | .010 | 517 (1.0) | 1584 (1.0) | .001 | 1336 (1.0) | 1584 (1.0) | .003 | 10 (0.9) | 1584 (1.0) | .005 |  |
|  | **Phentermine** | 64 (0.5) | 395 (0.3) | .042 | 92 (0.2) | 395 (0.3) | .016 | 280 (0.2) | 395 (0.3) | .007 | 10 (0.9) | 395 (0.3) | .091 |  |
|  | **Naltrexone** | 38 (0.3) | 234 (0.2) | .032 | 115 (0.2) | 234 (0.2) | .017 | 15 (0.0) | 30 (0.0) | .033 | 10 (0.9) | 234 (0.2) | .108 |  |
|  | **Orlistat** | 10 (0.1) | 30 (0.0) | .027 | 10 (0.0) | 30 (0.0) | .000 | 398 (0.3) | 234 (0.2) | .006 | 0 (0) | 30 (0.0) | .0194 |  |
| **Persons with potential health hazards related to socioeconomic and psychosocial circumstances** | | 237 (1.9) | 3740 (2.3) | .034 | 2892 (5.5) | 3740 (2.3) | .162 | 5263 (4.0) | 3740 (2.3) | .096 | 48 (4.5) | 3740 (2.3) | .120 |  |

Data are shown as median (SD) for continuous variables and number (%) for categorical variables. **BMI:** Body mass index. Orlistat, bupropion, naltrexone, and phentermine, topiramate were not included as propensity score matching covariates but are presented for additional information.

# **Supplementary Table S7:** Individual SGLT-2is baseline characteristics after propensity score matching.

|  | | | **Canagliflozin vs DPP-4i** | | | **Dapagliflozin vs DPP-4i** | | | **Empagliflozin vs DPP-4i** | | | **Ertugliflozin vs DPP-4i** | | |
| --- | --- | --- | --- | --- | --- | --- | --- | --- | --- | --- | --- | --- | --- | --- |
|  |  |  | **Exposure** | **Comparator (DPP-4i)** | **SMD** | **Exposure** | **Comparator (DPP-4i)** | **SMD** | **Exposure** | **Comparator (DPP-4i)** | **SMD** | **Exposure** | **Comparator (DPP-4i)** | **SMD** |
| **Number** | | | 12742 | 12742 | - | 41783 | 41783 | - | 73488 | 73488 | - | 1056 | 1056 | - |
| **Age at Index** | | | 57.9 (11.8) | 58.0 (13.3) | .006 | 64.0 (12.7) | 63.8 (14.7) | .008 | 63.0 (12.5) | 63.2 (14.5) | .014 | 54.8 (12.2) | 55.6 (13.7) | .062 |
| **Sex** | **Female** | | 5705 (44.8) | 5693 (44.7) | .002 | 18696 (44.8) | 18796 (45.0) | .005 | 34284 (46.7) | 34702 (47.2) | .011 | 499 (47.3) | 497 (47.1) | .004 |
|  | **Male** | | 7031 (55.2) | 7045 (55.3) | .002 | 23064 (55.2) | 22973 (55.0) | .004 | 39154 (53.3) | 38740 (52.7) | .011 | 556 (52.7) | 555 (52.6) | .002 |
|  | **Unknown sex** | | 10 (0.1) | 10 (0.1) | - | 23 (0.1) | 14 (0.0) | .010 | 50 (0.1) | 46 (0.1) | .002 | 10 (1.0) | 10 (1.0) | - |
| **Race** | **American Indian or Alaska Native** | | 64 (0.5) | 66 (0.5) | .002 | 233 (0.6) | 219 (0.5) | .005 | 461 (0.6) | 472 (0.6) | .002 | 11 (1.0) | 10 (1.0) | .010 |
|  | **Asian** | | 836 (6.6) | 859 (6.7) | .007 | 4245 (10.2) | 4423 (10.6) | .014 | 6486 (8.8) | 6709 (9.1) | .011 | 102 (9.7) | 96 (9.1) | .019 |
|  | **Black or African American** | | 2202 (17.3) | 2286 (17.9) | .017 | 8938 (21.4) | 8740 (20.9) | .012 | 15190 (20.0) | 15087 (20.5) | .003 | 218 (20.6) | 220 (20.8) | .005 |
|  | **Native Hawaiian or Other Pacific Islander** | | 96 (0.8) | 95 (0.8) | .001 | 323 (0.8) | 338 (0.8) | .004 | 772 (1.1) | 775 (1.1) | .000 | 43 (4.1) | 29 (2.8) | .073 |
|  | **White** | | 8446 (66.3) | 8351 (65.5) | .016 | 24235 (58.0) | 24184 (57.9) | .002 | 43293 (58.9) | 43067 (58.6) | .006 | 519 (49.2) | 525 (49.7) | .011 |
|  | **Other Race** | | 480 (3.8) | 494 (3.9) | .006 | 1564 (3.7) | 1611 (3.9) | .006 | 3078 (4.2) | 3021 (4.1) | .004 | 86 (8.1) | 87 (8.2) | .003 |
|  | **Unknown Race** | | 618 (4.9) | 591 (4.6) | .001 | 2245 (5.4) | 2268 (5.4) | .002 | 4208 (5.7) | 4357 (5.9) | .009 | 77 (7.3) | 93 (8.8) | .056 |
| **Ethnicity** | **Hispanic or Latino** | | 1116 (8.8) | 1049 (8.2) | .019 | 3340 (8.0) | 3319 (7.9) | .002 | 6286 (8.6) | 6245 (8.5) | .002 | 157 (14.9) | 158 (15.0) | .003 |
|  | **Not Hispanic or Latino** | | 9750 (76.5) | 9771 (76.7) | .004 | 30504 (73.0) | 30529 (73.1) | .001 | 53687 (73.1) | 53476 (72.8) | .006 | 705 (66.8) | 707 (67.0) | .004 |
|  | **Unknown Ethnicity** | | 1876 (14.7) | 1922 (15.1) | .010 | 7939 (19.0) | 7935 (19.0) | .000 | 13515 (18.4) | 13767 (18.7) | .009 | 194 (18.4) | 191 (18.1) | .007 |
| **BMI** | | | 34.5 (7.5) | 34.3 (7.5) | .021 | 32.1 (7.7) | 31.9 (7.6) | .035 | 32.3 (7.6) | 32.1 (7.6) | .034 | 33.3 (7.9) | 32.9 (7.8) | .062 |
| **BMI categories** | **<25 kg/m2** | | 1182 (9.3) | 1092 (8.6) | .025 | 8666 (20.7) | 8667 (20.7) | .000 | 14132 (19.2) | 14415 (19.6) | .010 | 177 (16.8) | 188 (17.8) | .028 |
|  | **25-30 kg/m2** | | 3602 (28.3) | 3631 (28.5) | .005 | 15802 (37.8) | 15740 (37.7) | .003 | 27085 (36.9) | 27067 (36.8) | .001 | 360 (34.1) | 373 (35.3) | .026 |
|  | **30-35 kg/m2** | | 4728 (37.1) | 4827 (37.9) | .016 | 14897 (35.7) | 14896 (35.7) | .000 | 26313 (35.8) | 26128 (35.6) | .005 | 365 (34.6) | 387 (36.7) | .044 |
|  | **35-40 kg/m2** | | 3624 (28.4) | 3531 (27.7) | .016 | 9490 (22.7) | 9496 (22.7) | .000 | 16841 (22.9) | 16598 (22.6) | .008 | 259 (24.5) | 259 (24.5) | - |
|  | **40-45 kg/m2** | | 2148 (16.9) | 2138 (16.8 | .002 | 5035 (12.1) | 4990 (11.9) | .003 | 8911 (12.1) | 8781 (12.0) | .005 | 155 (14.7) | 165 (15.6) | .026 |
|  | **45-50 kg/m2** | | 1003 (7.9) | 1066 (8.4) | .018 | 2432 (5.8) | 2343 (5.6) | .009 | 4252 (5.8) | 4185 (5.7) | .004 | 80 (7.6) | 82 (7.8) | .007 |
|  | **50-55 kg/m2** | | 446 (3.5) | 471 (3.7) | .011 | 1169 (2.8) | 1103 (2.6) | .010 | 1925 (2.6) | 1945 (2.7) | .002 | 41 (3.9) | 38 (3.6) | .015 |
|  | **>55 kg/m2** | | 276 (2.2) | 276 (2.2) | - | 766 (1.8) | 723 (1.7) | .008 | 1272 (1.7) | 1279 (1.7) | .001 | 24 (2.3) | 18 (1.7) | .041 |
| **HbA1c** | | | 8.5 (1.9) | 8.5 (1.9) | .003 | 7.9 (1.9) | 8.0 (1.9) | .059 | 8.1 (1.9) | 8.2 (1.9) | .010 | 8.8 (2.2) | 8.7 (2.2) | .027 |
| **HbA1c categories** | **<6.5 %** | | 2148 (16.9) | 2166 (17.0) | .004 | 13018 (31.2) | 12800 (30.6) | .011 | 18009 (24.5) | 18073 (24.6) | .002 | 207 (19.6) | 229 (21.7) | .051 |
|  | **6.5-7.5 %** | | 5081 (39.9) | 5095 (40.0) | .002 | 17647 (42.2) | 17672 (42.3) | .001 | 31220 (42.5) | 31209 (42.5) | .000 | 352 (33.3) | 360 (34.1) | .016 |
|  | **7.5-8.5 %** | | 5020 (39.4) | 4941 (38.8) | .013 | 11881 (28.4) | 12081 (28.9) | 0.011 | 24494 (33.3) | 24400 (33.2) | .003 | 319 (30.2) | 321 (30.4) | .004 |
|  | **8.5-9.5 %** | | 3282 (25.8) | 3245 (25.5) | .007 | 6754 (16.2) | 6953 (16.6) | .013 | 14668 (20.0) | 14736 (20.1) | .002 | 232 (22.0) | 247 (23.4) | .034 |
|  | **9.5-10.5 %** | | 2046 (16.1) | 2003 (15.7) | .009 | 3992 (9.6) | 4056 (9.7) | .005 | 8762 (11.9) | 8695 (11.8) | .003 | 169 (16.0) | 160 (15.2) | .024 |
|  | **10.5-11.5 %** | | 1364 (10.7) | 1400 (11.0) | .009 | 2624 (6.3) | 2658 (6.4) | .003 | 5624 (7.7) | 5649 (7.7) | .001 | 119 (11.3) | 110 (10.4) | .027 |
|  | **>11.5 %** | | 1369 (10.7) | 1351 (10.6) | .005 | 3427 (8.2) | 3543 (8.5) | .010 | 7196 (9.8) | 7114 (9.7) | .004 | 181 (17.1) | 186 (17.6) | .012 |
| **Past medical history** | **Hypertensive diseases** | | 9510 (74.6) | 9566 (75.1) | .010 | 34018 (81.4) | 33718 (80.7) | .018 | 57661 (78.5) | 57496 (78.2) | .005 | 720 (68.2) | 728 (68.9) | .016 |
|  | **Ischemic heart diseases** | | 2210 (17.3) | 2169 (17.0) | .009 | 13824 (33.1) | 13390 (32.1) | .022 | 19445 (26.5) | 19054 (25.9) | .012 | 149 (14.1) | 121 (11.5) | .079 |
|  | **Type 2 diabetes mellitus with kidney complications** | | 1491 (11.7) | 1487 (11.7) | .001 | 10572 (25.3) | 10264 (24.6) | .017 | 14930 (20.3) | 14963 (20.4) | .001 | 131 (12.4) | 123 (11.7) | .023 |
|  | **Type 2 diabetes mellitus with neurological complications** | | 1599 (12.6) | 1564 (12.3) | .008 | 6355 (15.2) | 6248 (15.0) | .007 | 10694 (14.6) | 10748 (14.6) | .002 | 153 (14.5) | 175 (16.6) | .058 |
|  | **Nicotine dependence** | | 1227 (9.6) | 1211 (9.5) | .004 | 5202 (12.5) | 5162 (12.4) | .003 | 8522 (11.6) | 8411 (11.5) | .005 | 178 (16.9) | 182 (17.2) | .010 |
|  | **Alcohol dependence** | 119 (0.9) | 114 (0.9) | .004 | 509 (1.2) | 513 (1.2) | .001 | 869 (1.2) | 832 (1.1) | .005 | 25 (2.4) | 20 (1.9) | .033 |  |
|  | **Cerebrovascular diseases** | | 699 (5.5) | 679 (5.3) | .007 | 5225 (12.5) | 5108 (12.2) | .009 | 7700 (10.5) | 7687 (10.5) | .001 | 72 (6.8) | 64 (6.1) | .031 |
|  | **Type 2 diabetes mellitus with ophthalmic complications** | | 615 (4.8) | 618 (4.9) | .001 | 2121 (5.1) | 2156 (5.2) | .004 | 3966 (5.4) | 3898 (5.3) | .004 | 46 (4.4) | 44 (4.2) | .009 |
|  | **Atherosclerosis** | | 343 (2.7) | 332 (2.6) | .005 | 2850 (6.8) | 2742 (6.6) | .010 | 3933 (5.4) | 3874 (5.3) | .004 | 24 (2.3) | 22 (2.1) | .013 |
|  | **Personal history of nicotine dependence** | | 1288 (10.1) | 1258 (9.9) | .008 | 7306 (17.5) | 7119 (17.0) | .012 | 11319 (15.4) | 11168 (15.2) | .006 | 134 (12.7) | 115 (10.9) | .056 |
|  | **Peripheral vascular disease, unspecified** | | 375 (2.9) | 338 (2.7) | .018 | 2612 (6.3) | 2535 (6.1) | .008 | 3757 (5.1) | 3742 (5.1) | .001 | 28 (2.7) | 35 (3.3) | .039 |
|  | **Alcohol related disorders** | | 272 (2.1) | 268 (2.1) | .002 | 1267 (3.0) | 1245 (3.0) | .003 | 2000 (2.7) | 1936 (2.6) | .005 | 44 (4.2) | 38 (3.6) | .029 |
| **Medications** | **Metformin** | | 8194 (64.3) | 8092 (63.5) | .017 | 20165 (48.3) | 20510 (49.1) | .017 | 41980 (57.1) | 42151 (57.4) | .005 | 726 (68.8) | 742 (70.3) | .033 |
|  | **Insulin** | | 4122 (32.4) | 3985 (31.3) | .023 | 18507 (44.3) | 18384 (44.0) | .006 | 28360 (38.6) | 28455 (38.7) | .003 | 418 (39.6) | 382 (36.2) | .070 |
|  | **Antihypertensives** | | 1062 (8.3) | 1021 (8.0) | .012 | 8413 (20.1) | 8109 (19.4) | .018 | 10807 (14.7) | 10897 (14.8) | .003 | 112 (10.6) | 106 (10.0) | .019 |
|  | **Sulfonylureas** | | 0.049 (28.7) | 3629 (28.5) | .004 | 7245 (17.3) | 7385 (17.7) | .009 | 16227 (22.1) | 16414 (22.3) | .006 | 278 (26.3) | 310 (29.4) | .068 |
|  | **Lipid modifying agents** | | 7598 (59.6) | 7548 (59.2) | .008 | 28803 (68.9) | 28568 (68.4) | .012 | 48823 (66.4) | 48578 (66.1) | .007 | 644 (61.0) | 666 (63.1) | .043 |
|  | **Bupropion** | | 438 (3.4) | 417 (3.3) | .009 | 1206 (2.9) | 1209 (2.9) | .000 | 2304 (3.1) | 2097 (2.9) | .017 | 38 (3.6) | 37 (3.5) | .005 |
|  | **Topiramate** | | 139 (1.1) | 151 (1.2)) | .009 | 403 (1.0) | 465 (1.1) | .015 | 775 (1.1) | 818 (1.1) | .006 | 10 (1.0) | 19 (1.8) | .073 |
|  | **Phentermine** | | 64 (0.5) | 35 (0.3) | .037 | 81 (0.2) | 81 (0.2) | - | 202 (0.3) | 160 (0.2) | .012 | 10 (1.0) | 10 (1.0) | - |
|  | **Naltrexone** | | 38 (0.3) | 27 (0.2) | .017 | 87 (0.2) | 98 (0.2) | .006 | 203 (0.3) | 155 (0.2) | .013 | 10 (1.0) | 10 (1.0) | - |
|  | **Orlistat** | | 10 (0.1) | 10 (0.1) | - | 10 (0.0) | 10 (0.0) | - | 12 (0.0) | 10 (0.0) | .002 | 0 (0) | 10 (1.0) | .138 |
| **Persons with potential health hazards related to socioeconomic and psychosocial circumstances** | | | 237 (1.9) | 231 (1.8) | .004 | 1813 (4.3) | 1774 (4.3) | .005 | 2609 (3.6) | 2499 (3.4) | .008 | 47 (4.5) | 41 (3.9) | .028 |

Data are shown as median (SD) for continuous variables and number (%) for categorical variables. **BMI:** Body mass index**.** Orlistat, bupropion, naltrexone, and phentermine, topiramate were not included as propensity score matching covariates but are presented for additional information.

**Supplementary Figure S1:** Kaplan–Meier curves for cancer outcomes by treatment group (SGLT-2i vs DPP-4i) over follow-up.


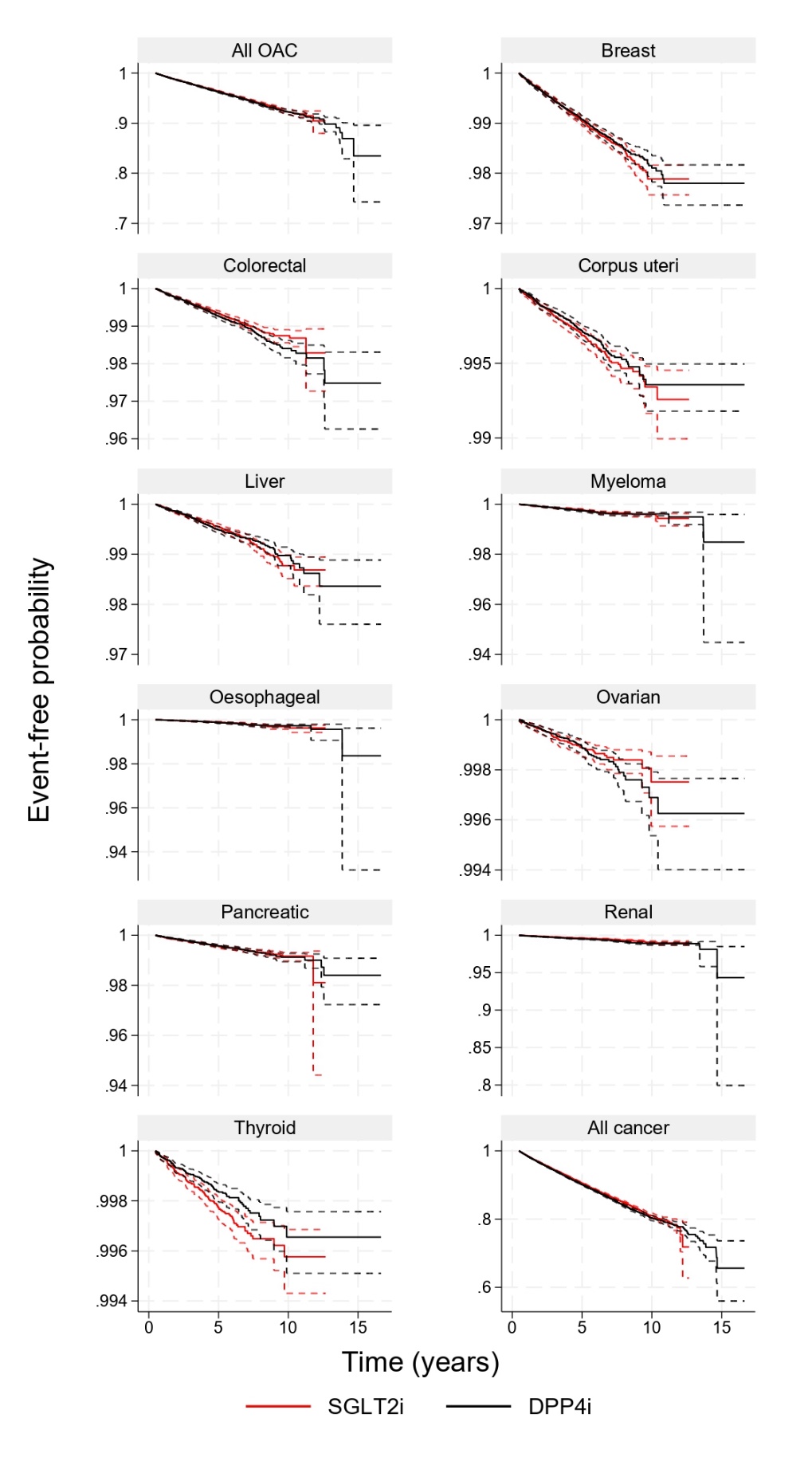


**Supplementary Figure S2:** Hazard ratio of obesity-associated cancers in individuals with type 2 diabetes treated with canagliflozin, dapagliflozin, empagliflozin or ertugliflozin, as compared with DPP-4i.


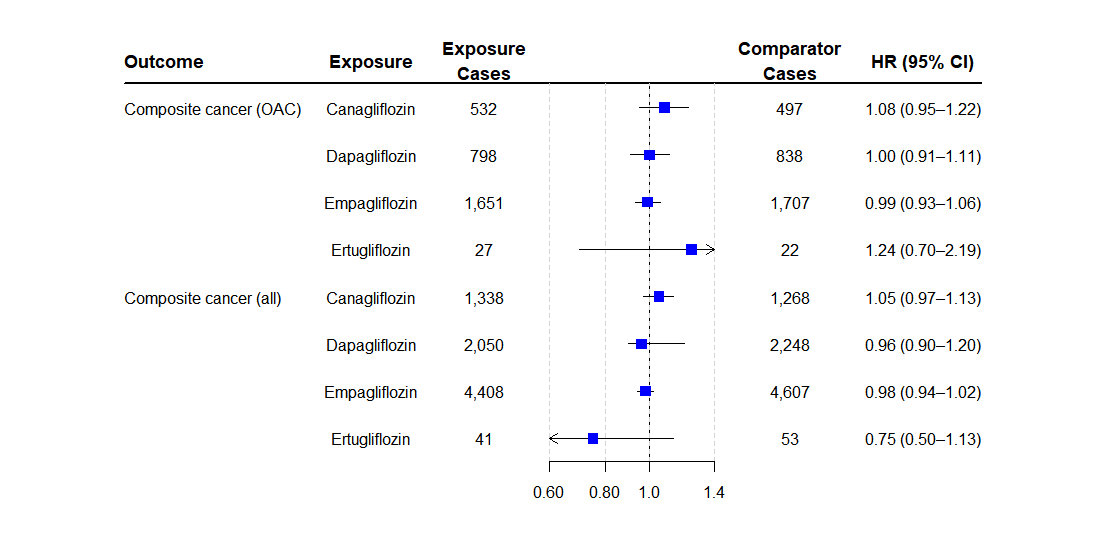


**HR**: Harzard Ratio; **CI**: Confidence Interval. The cohorts included individuals with and without overweight/obesity

**Supplementary Figure S3:** Hazard ratio of sensitivity analysis excluding individuals with GLP-1 RA or tirzepatide use following SGLT-2i/DPP-4i initiation.


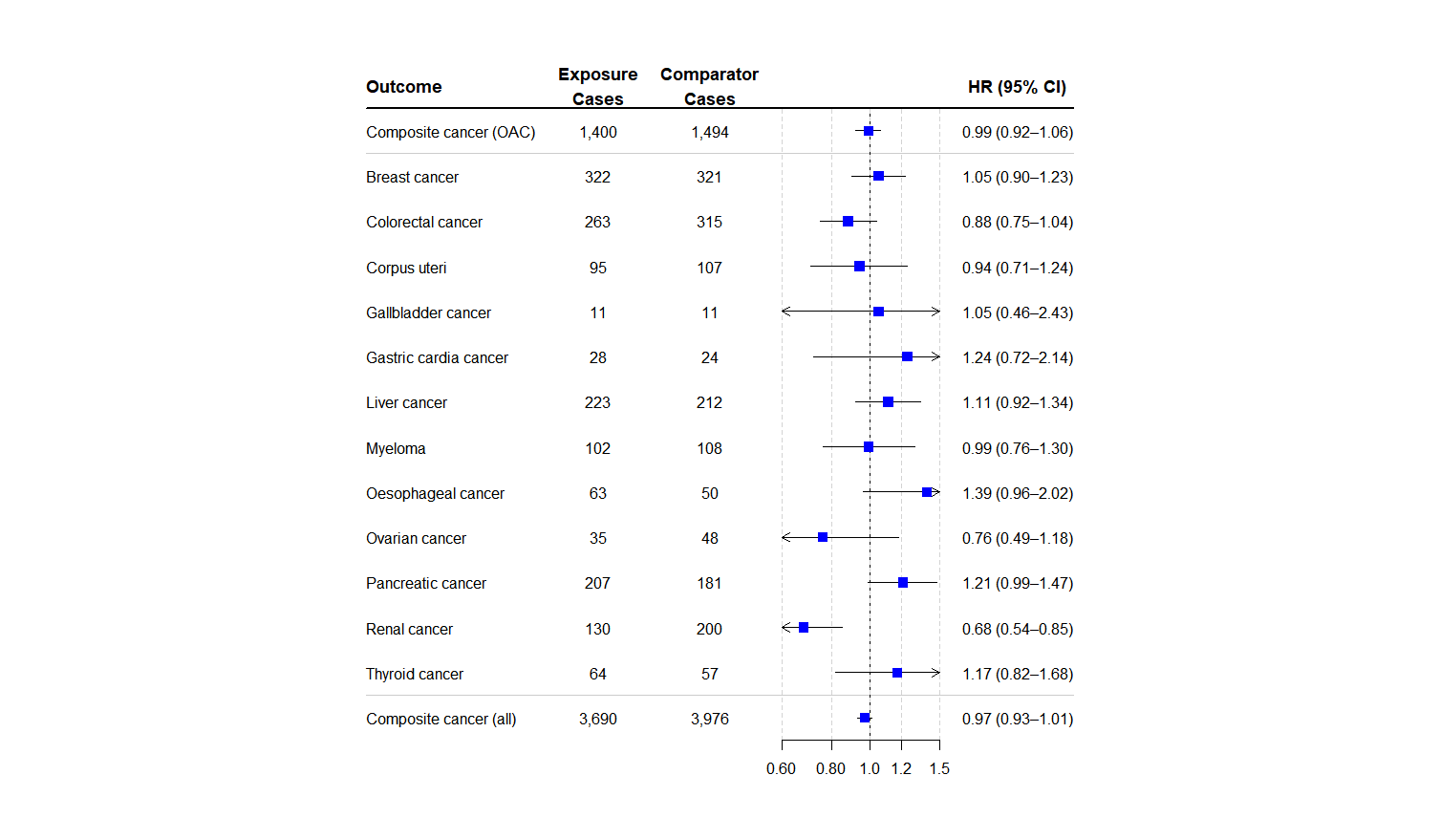


**HR**: Harzard Ratio; **CI**: Confidence Interval. The cohorts included individuals in the overall population.

**Supplementary Figure S4:** Hazard ratio of sensitivity analysis restricted to individuals with repeated SGLT-2i or DPP-4i use at least six months after initial initiation


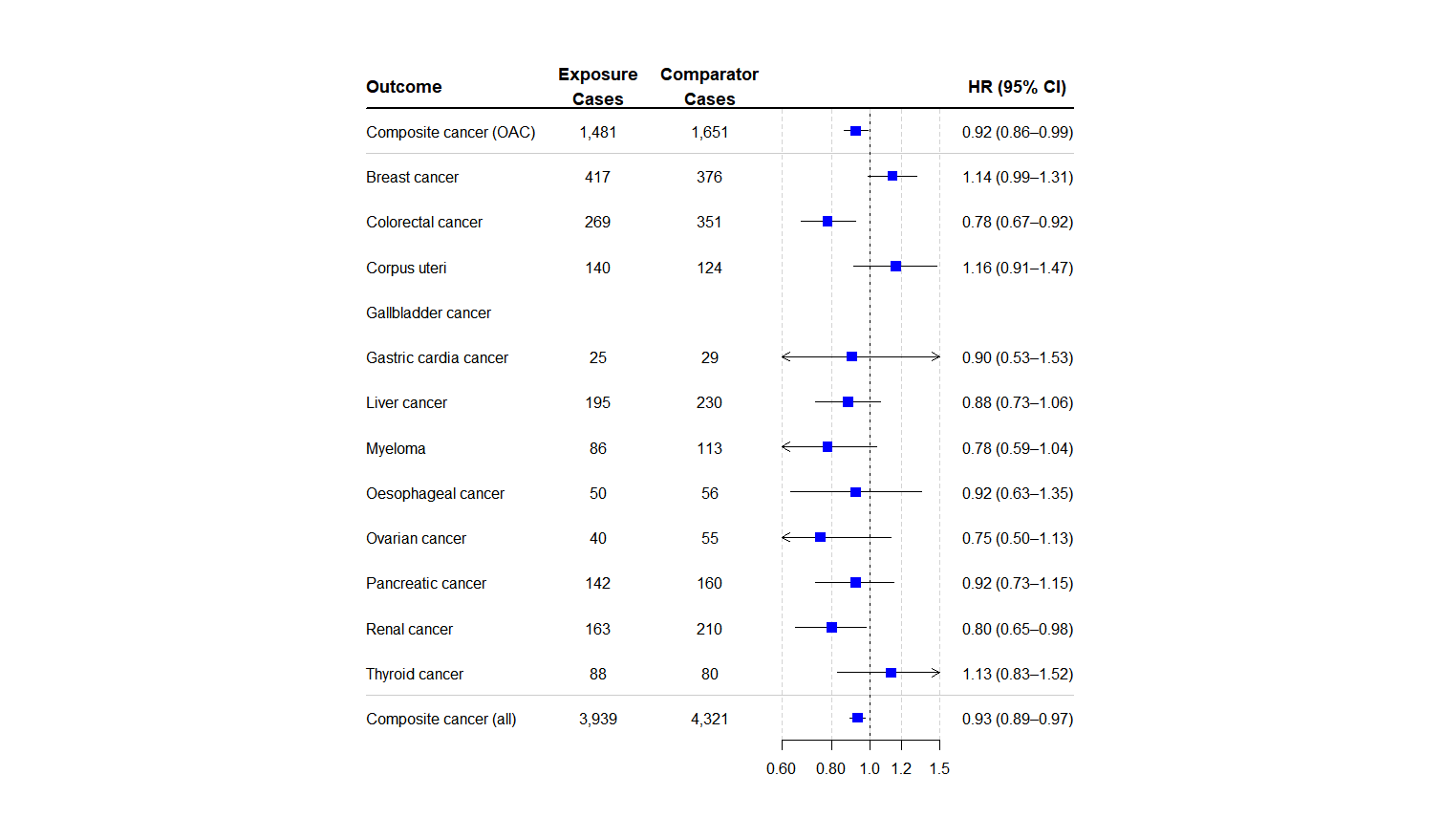


**HR**: Harzard Ratio; **CI**: Confidence Interval. The cohorts included individuals in the overall population.

#

# **RECORD checklist**

**The RECORD statement – checklist of items, extended from the STROBE statement, that should be reported in observational studies using routinely collected health data.**

|  | **Item No.** | **STROBE items** | **Location in manuscript where items are reported** | **RECORD items** | **Location in manuscript**  **where items are reported** |
| --- | --- | --- | --- | --- | --- |
| **Title and abstract** | | | | | |
|  | 1 | (a) Indicate the study’s design with a commonly used term in the title or the abstract (b)  Provide in the abstract an informative and balanced  summary of what was done and what was found | Title and abstract page 1-3 | RECORD 1.1: The type of data used should be specified in the title or abstract. When possible, the name of  the databases used should be included.  RECORD 1.2: If applicable, the geographic region and timeframe within which the study took place should be reported in the title or abstract.  RECORD 1.3: If linkage between  databases was conducted for the study, this should be clearly stated in the title or abstract. | Title and abstract page 1-3 |
| **Introduction** | | | | | |
| Background rationale | 2 | Explain the scientific background and rationale for the investigation being reported | Introduction page 4-5 |  |  |
| Objectives | 3 | State specific objectives, including any prespecified hypotheses | Introduction page 4-5 |  |  |
| **Methods** | | | | | |
| Study Design | 4 | Present key elements of study design early in the paper | Materials and Methods page 6-10 |  |  |
| Setting | 5 | Describe the setting, locations, and relevant dates, including  periods of recruitment, exposure, follow-up, and data collection | Materials and Methods “Study Population” section page 6-7 |  |  |
| Participants | 6 | *(a) Cohort study* - Give the | Materials and Methods “Study Population” section page 6-7 | RECORD 6.1: The methods of study | Materials Methods “Data Source” and “Study |

|  |  | eligibility criteria, and the  sources and methods of selection of participants. Describe  methods of follow-up  *Case-control study* - Give the eligibility criteria, and the  sources and methods of case ascertainment and control selection. Give the rationale for the choice of cases and controls *Cross-sectional study* - Give the eligibility criteria, and the  sources and methods of selection of participants  *(b) Cohort study* - For matched studies, give matching criteria and number of exposed and unexposed  *Case-control study* - For matched studies, give matching criteria and the number of  controls per case |  | population selection (such as codes or algorithms used to identify subjects) should be listed in detail. If this is not possible, an explanation should be provided.  RECORD 6.2: Any validation studies of the codes or algorithms used to select the population should be referenced. If validation was conducted for this study and not published elsewhere, detailed methods and  results should be provided.  RECORD 6.3: If the study involved  linkage of databases, consider use of a flow diagram or other graphical display to demonstrate the data linkage process, including the number of  individuals with linked data at each stage. | Population” section page 6-7 |
| --- | --- | --- | --- | --- | --- |
| Variables | 7 | Clearly define all outcomes, exposures, predictors, potential confounders, and effect modifiers. Give diagnostic criteria, if applicable. | Materials and Methods “Study Population”, “Outcomes”, and “Confounding factors” sections page 6-8 | RECORD 7.1: A complete list of codes and algorithms used to classify exposures, outcomes, confounders, and effect modifiers should be provided. If these cannot be reported, an explanation should be provided. | Materials and Methods “Study Population”, “Outcomes”, and “Confounding factors” sections page 6-8 |
| Data sources/ measurement | 8 | For each variable of interest,  give sources of data and details of methods of assessment (measurement).  Describe comparability of assessment methods if there is more than one group | Materials and Methods “Study Population”, “Outcomes”, and “Confounding factors” sections page 6-8 |  |  |
| Bias | 9 | Describe any efforts to address potential sources of bias | In sub analysis of individuals with or without overweight/obesity, interaction p-values for overweight/obesity status and SGLT-2i use with respect to cancer risk were manually calculated. |  |  |

| Study size | 10 | Explain how the study size was  arrived at | Materials and Methods “Study Population” section page 6-7 |  |  |
| --- | --- | --- | --- | --- | --- |
| Quantitative variables | 11 | Explain how quantitative  variables were handled in the analyses. If applicable, describe which groupings were chosen, and why | Materials and Methods “Statistical analysis” section page 9-10 |  |  |
| Statistical methods | 12 | 1. Describe all statistical methods, including those used to control for confounding 2. Describe any methods used to examine subgroups and   interactions   1. Explain how missing data were addressed 2. *Cohort study* - If applicable, explain how loss to follow-up was addressed   *Case-control study* - If applicable, explain how matching of cases and controls was addressed  *Cross-sectional study* - If applicable, describe analytical methods taking account of sampling strategy   1. Describe any sensitivity analyses | Materials and Methods “Statistical analysis” section page 9-10 |  |  |
| Data access and cleaning methods |  | .. |  | RECORD 12.1: Authors should describe the extent to which the  investigators had access to the database population used to create the study population.  RECORD 12.2: Authors should provide information on the data  cleaning methods used in the study. | Materials and Methods “Statistical analysis” section page 9-10 |
| Linkage |  | .. |  | RECORD 12.3: State whether the | Materials and Methods “Data Source” section |

|  |  |  |  | study included person-level, institutional-level, or other data linkage across two or more databases. The  methods of linkage and methods of linkage quality evaluation should be provided. | page 6 |
| --- | --- | --- | --- | --- | --- |
| **Results** | | | | | |
| Participants | 13 | 1. Report the numbers of   individuals at each stage of the study (*e.g.*, numbers potentially eligible, examined for eligibility, confirmed eligible, included in  the study, completing follow-up, and analysed)   1. Give reasons for non- participation at each stage. 2. Consider use of a flow diagram | NA | RECORD 13.1: Describe in detail the selection of the persons included in the study (*i.e.,* study population selection) including filtering based on data quality, data availability and linkage. The selection of included persons can be described in the text and/or by  means of the study flow diagram. | NA |
| Descriptive data | 14 | (a) Give characteristics of study participants (*e.g*., demographic, clinical, social) and information on exposures and potential confounders  (b) Indicate the number of participants with missing data for each variable of interest (c) *Cohort study* - summarise follow-up time (*e.g*., average and total amount) | Table 1, Figure 1, Table S4 |  |  |
| Outcome data | 15 | *Cohort study* - Report numbers  of outcome events or summary  measures over time | Figure 2 |  |  |

|  |  |  |  |  |  |
| --- | --- | --- | --- | --- | --- |
| Main results | 16 | (a) Give unadjusted estimates  and, if applicable, confounder-  adjusted estimates and their  precision (e.g., 95% confidence  interval). Make clear which  confounders were adjusted for  and why they were included  (b) Report category boundaries  when continuous variables were  categorized  (c) If relevant, consider  translating estimates of relative  risk into absolute risk for a  meaningful time period | Figure 2 |  |  |
| Other analyses | 17 | Report other analyses done—e.g., analyses of  subgroups and interactions, and sensitivity analyses | Figure 3, Figure S2-4 |  |  |
| **Discussion** | | | | | |
| Key results | 18 | Summarise key results with reference to study objectives | Discussion page 16 - 17 |  |  |
| Limitations | 19 | Discuss limitations of the study, taking into account sources of potential bias or imprecision.  Discuss both direction and  magnitude of any potential bias | Discussion “Strengths and limitations” section page 20 - 22 | RECORD 19.1: Discuss the  implications of using data that were not created or collected to answer the  specific research question(s). Include discussion of misclassification bias, unmeasured confounding, missing data, and changing eligibility over time, as they pertain to the study being reported. | Discussion “Strengths and limitations” section page 20 - 22 |
| Interpretation | 20 | Give a cautious overall interpretation of results considering objectives, limitations, multiplicity of analyses, results from similar studies, and other relevant  evidence | Discussion page 16 - 22 |  |  |

| Generalisability | 21 | Discuss the generalisability (external validity) of the study  results | Discussion “Comparison with prior studies”, and “Mechanisms related to anti-cancer effects of SGLT-2i”, sections page 17 - 20 |  |  |
| --- | --- | --- | --- | --- | --- |
| **Other Information** | | | | | |
| Funding | 22 | Give the source of funding and the role of the funders for the present study and, if applicable, for the original study on which the present article is based | Acknowledgement page 22 |  |  |
| Accessibility of protocol, raw data, and programming  code |  | .. |  | RECORD 22.1: Authors should  provide information on how to access any supplemental information such as the study protocol, raw data, or programming code. | Materials and Methods “Study Population”, “Outcomes”, and “Confounding factors” sections page 6-8 |

*Reference: Benchimol EI, Smeeth L, Guttmann A, Harron K, Moher D, Petersen I, Sørensen HT, von Elm E, Langan SM, the RECORD Working Committee. The REporting of studies Conducted using Observational Routinely-collected health Data (RECORD) Statement. *PLoS Medicine* 2015; in press.

*Checklist is protected under Creative Commons Attribution ([CC BY](http://creativecommons.org/licenses/by/4.0/)) license.
